# Drastic Impact of Donor Substituents on Xanthenes in the PDT of Glioblastoma

**DOI:** 10.1021/jacsau.5c00738

**Published:** 2025-10-01

**Authors:** O. Karaman, E. Yesilcimen, M. Forough, Z. Elmazoglu, G. Gunbas

**Affiliations:** † Department of Chemistry, 52984 Middle East Technical University, Ankara 06800, Turkiye; ‡ Faculty of Pharmacy, Ankara Medipol University, Ankara 06050, Turkiye

**Keywords:** photodynamic therapy, glioblastoma, xanthane-based photosensitizers, near-infrared absorption, selenium-containing dyes

## Abstract

Even though significant progress has been made in treating various cancer types, brain cancers lag drastically. Several factors contribute, led by operational difficulties, the blood-brain barrier, and the tendency of recurrence. Alternative therapies are needed, and photodynamic therapy (PDT) offers several advantages. However, PDT has rarely been explored for brain cancers since, for achieving near-infrared range activation, photosensitizers should have longer conjugation lengths and thus higher molecular weight, which then limits blood-brain barrier penetration. Here, we describe the syntheses and PDT action of two new selenium-containing xanthane-based photosensitizers (**NSeMorph** and **NSeAze**) that show absorption over 650 nm with molecular weights lower than 420 g/mol. It has been demonstrated that both **NSeMorph** and **NSeAze** showed PDT activity against glioblastoma cell lines (U87MG and U118MG), and more interestingly, the efficacy and selectivity of the photosensitizers were significantly different depending on the donor side groups. **NSeMorph**, utilizing morpholine donors, showed IC_50_ values of 15.8 μM for U87MG and 8.0 μM for U118MG cell lines. Surprisingly, the IC_50_ was not reached at a 20 μM concentration in the healthy cell line (L929), indicating the selective nature of **NSeMorph** even though no activation-based cage groups or targeting groups were present. Upon switching the donor units to azetidine, IC_50_ values of 456 nM for U87MG and 461 nM for U118MG cell lines were achieved; to the best of our knowledge, these are the lowest IC_50_ values reported in literature against glioblastoma (U87MG and U118MG) that combine NIR absorption in aqueous media and low molecular weight (<400 g/mol). Additionally, **NSeAze** showed one of the highest phototoxicity indices, the ratio of cell viabilities under dark and light conditions, showing the remarkable activity of **NSeAze** under illumination. Overall, this study represents one of the first examples of the drastic effect on PDT action by altering only the donor side groups of xanthene-based dyes.

## Graphical abstract

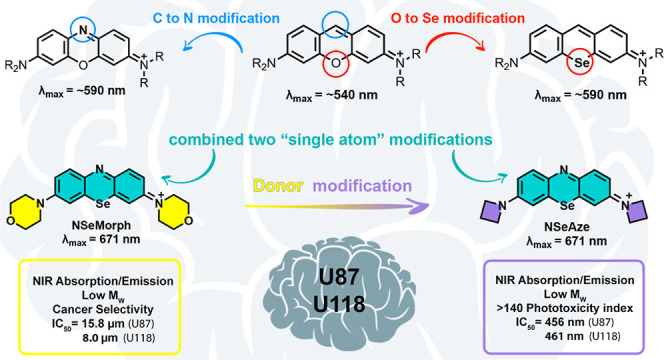

## Introduction

Cancer remains one of the leading causes of death, with 20 million new cancer cases and 9.7 million deaths in the year 2022.^1^ Even though significant advancements were achieved, which translated into much improved survival rates in particular cancer types, especially in breast and prostate cancers, mortality and 5 year survival rates are mostly stagnant among brain cancer patients.^2^ The high incidence of inoperable tumors and scarcity of effective medication due to the blood-brain barrier (BBB) are the leading causes for this bleak outlook.^3^ Several new treatment modalities are evolving in the cancer patient-care arena in addition to conventional methods of chemotherapy and radiation. Immunotherapy is making its mark in the field of cancer, with approaches ranging from immune checkpoint inhibitors^4–6^ to T-cell transfer therapy^7–9^ and immune system modulators.^10–13^ However, resistance to immunotherapy and side effects related to highly active immune systems prove challenging.^14,15^

Photodynamic Therapy (PDT) has attracted considerable interest in recent years due to its minimally invasive approach and fewer side effects compared to conventional treatments.^16^ In standard PDT, a photosensitizer (PS) in its ground state absorbs light of a specific wavelength, leading to its excitation. Through intersystem crossing (ISC), the PS transitions from the singlet excited state to the triplet excited state. Upon interacting with molecular oxygen (^3^O_2_), reactive oxygen species (ROS), mainly singlet oxygen (^1^O_2_), are generated, which exert cytotoxic effects on cells at the treatment site.^17–19^ Although various approaches are being pursued for bringing the advantages of PDT to brain cancer treatment, the volume of work is still quite limited, especially compared to other cancer types.^20,21^ Clinically, there are only a handful of studies, and although initial results are promising, much progress is needed toward improving survival rates.^22,23^ Main agent design involves small molecule drugs that have evolved from first-generation porphyrins to a wide range of agents as well as modified nano/carrier systems, which, in most cases, also incorporate a small molecule PDT agent. Several requirements exist for such small molecules, including, but not limited to, water solubility, photostability, and absorption in the NIR region for the treatment of deeper tumors.^24,25^ Among next-generation small molecule PDT agents satisfying these conditions, xanthene-based systems are becoming increasingly popular. However, the vast majority of work for their use is in imaging studies where they demonstrated outstanding success.^26–28^ For brain tumors, a PDT agent that exhibits strong absorption in the NIR region (>650 nm) and has a low molecular weight (MW) (400 g/mol^29^ or 500 g/mol^30^) for BBB penetration is required. Unfortunately, these are contradicting characteristics, since the general methodology to shift the absorption to the infrared is to extend the conjugation in the molecules, which ultimately results in higher molecular weights.^31,32^

In our pursuit of realizing an NIR-absorbing PDT agent with small molecular weights, we focused on single-atom modifications of fluorescein, rhodamine, or rhodol-type dyes to achieve both red-shifted absorption and singlet oxygen generation capability, while maintaining molecular weights under 500 g/mol.^33^ Herein, we report the design, synthesis, and PDT action potential of two NIR-absorbing dyes based on two single-atom modifications of the rhodamine core, **NSeMorph** and **NSeAze** (Scheme 1).

**Scheme 1:**
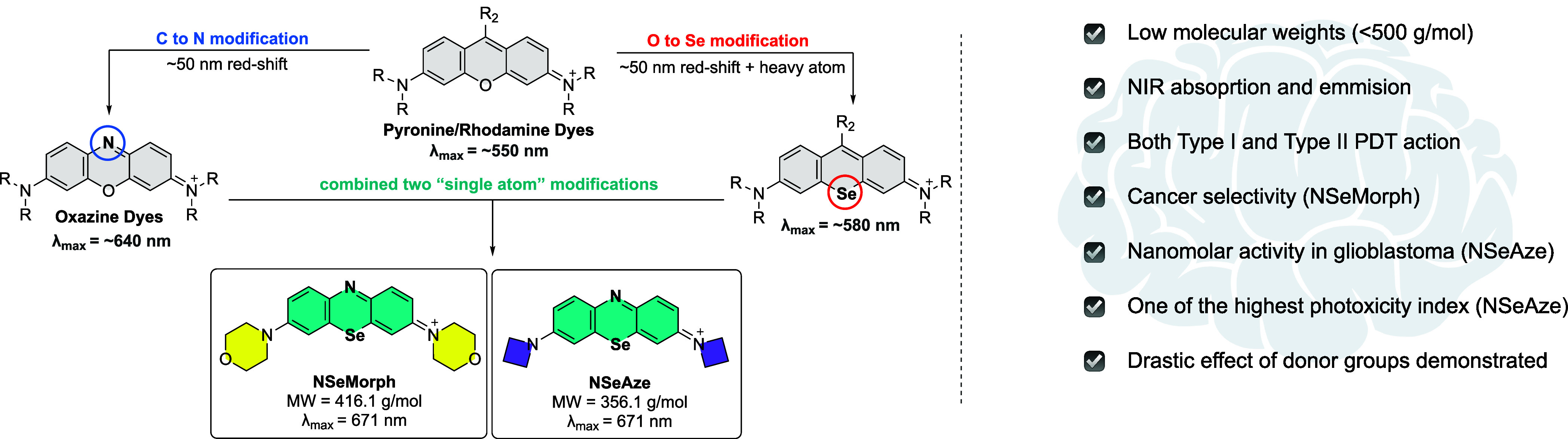
Design Principles of NIR Photosensitizers with Low Molecular Weights

**NSeMorph** and **NSeAze** both exhibit strong absorption beyond 650 nm while maintaining relatively low molecular weights (415 and 355 g/mol, cationic parts). Our findings reveal that both compounds exhibit potent PDT activity against glioblastoma cell lines (U87MG and U118MG), with notable differences in efficacy and selectivity arising solely from variations in the donor-side substituents. Specifically, while **NSeMorph** exhibited apparent selectivity for cancer cell lines despite the absence of activation-dependent caging or targeting moieties, in contrast, **NSeAze** showed dramatically enhanced potency, with IC_50_ values in the nanomolar range, albeit with no selectivity. However, **NSeAze** also exhibited one of the highest recorded phototoxicity indices—defined as the ratio of cell viability in the dark to that under irradiation—for glioblastoma cell lines, highlighting its exceptional light-triggered cytotoxicity and its safety at high concentrations in the dark.

To the best of our knowledge, **NSeAze** exhibits the lowest IC_50_ reported in the literature against glioblastoma cells, which combines NIR absorption in aqueous media and a low molecular weight (Table S3).^34–60^ Exceptional PDT action in breast cancer cell lines and the effect of substituents on the phototoxicity index (PI) have been demonstrated with similar selenium-bearing cores recently.^61^ However, this work represents the first demonstration of how subtle modulation of donor side groups in xanthene scaffolds can elicit profound changes in both PDT efficacy and selectivity, specifically in glioblastoma.

## Results and Discussion

### Design and Synthesis of NIR Photosensitizers

For the realization of an NIR-absorbing PDT agent with low molecular weights, we focused on two single-atom modifications of rhodamine dyes to achieve both red-shifted absorption and singlet oxygen generation capability while maintaining MW’s under 500 g/mol (Scheme 1). Regarding the MW calculations, it is standard practice in chemical conventions to include counterions. However, for central nervous system (CNS) drug penetration criteria, particularly across the BBB, the MW is often considered for the free base, as this is typically the primary form facilitating passive diffusion. For predominantly protonated or cationic drugs, penetration frequently occurs via solute carrier (SLC) proteins, such as organic cation transporters (OCTs).^62^ In such cases, the cationic moiety is the critical species for transport, making its MW highly relevant. This approach aligns with observed BBB penetration mechanisms for various cationic compounds where the cation itself is the key transported entity. Furthermore, while hydrophobic counterions can facilitate cell penetration through ion-pairing, hydrophilic anions like chloride and iodide are generally assumed to dissociate fully in aqueous solutions, leading to separate species rather than stable ion pairs influencing permeation.^63,64^

Literature shows that carbon-to-nitrogen modification in rhodamines results in oxazine cores with a red shift of around 90 nm, while oxygen-to-selenium modification results in a smaller but noticeable shift of around 30 nm (Scheme 1).^65–69^ The critical question here is whether the combination of these modifications results in an additive effect on absorption maxima, and our studies, as well as results from others on similar combined modifications, suggest an additive nature.^65,70,71^ Finally, the two donor side groups, morpholine and azetidine, were selected for the design of photosensitizers **NSeMorph** and **NSeAze**, respectively. Donor group modifications are common in the literature; however, these are generally investigated for their effect on fluorescence quantum yields and subsequent imaging studies.^16,72^ Utilization of fused systems as donors in shifting absorption and emission maxima is also well documented.^66,73–80^ Here, however, the primary motivation was to see if the side donors would impart notable differences in cellular localization and singlet-oxygen quantum yields, which would then impart critical differences in PDT action. The drastic difference observed, however, was not foreseen (Scheme 1). Both structures exhibit high biocompatibility, drug likeness, and compliance with Lipinski rules for potential BBB penetration, as determined by SwissADME calculations.^81^ In addition, the lightBBB tool developed by Shaker et al., which builds on large data sets and revealed 90% accuracy related to BBB permeability, predicts that both **NSeAze** and **NSeMorph** are BBB penetrable.^82^ It is also important to note here that methylene blue, a structurally similar compound, is also a known CNS drug and penetrates through the BBB.^83,84^

The synthetic route for the NIR photosensitizers is given in Scheme 2. Treatment of diphenylamine (**1**) with molecular selenium and selenium oxide in the presence of iodine gave compound **2**. Protection with PMB–Cl, followed by bromination in the presence of NBS, gave dibromo derivative **4**. Buchwald–Hartwig coupling with morpholine gave compound **5**, and oxidative deprotection with molecular iodine gave the title compound **NSeMorph** (34% yield over five steps). For the synthesis of **NSeAze**, compound **4** was coupled with azetidine in the presence of a palladium catalyst to get compound **6**. Following the same oxidative deprotection above yielded the title compound **NSeAze** (26% yield over five steps). This modular and efficient synthetic strategy paves the way for creating a wide range of NIR dyes with diverse side donor units—making it possible to explore how these variations impact photophysical properties and PDT efficacy.

**Scheme 2:**
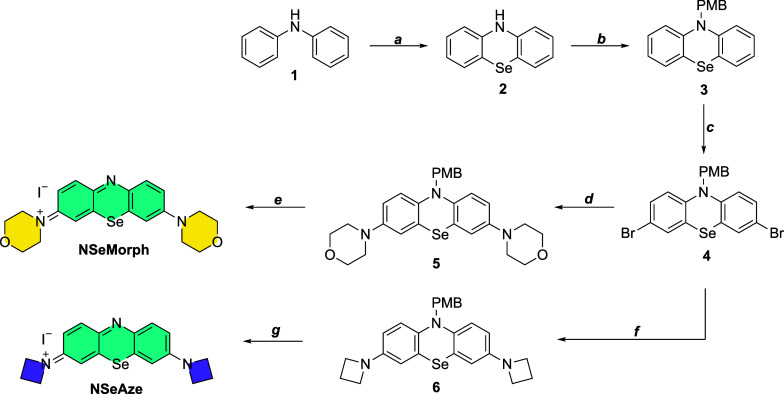
Reagents and Conditions: (a) Se, SeO_2_, I_2_, Sulfolane, 5 h, 150 °C, **75%**, (b); (1) NaH, DMF, rt, 30 min, (2) PMB–Cl, 80 °C, 16 h, **95%**, (c); NBS, DMF, rt, 16 h, **61%**, (d); Morpholine, Pd­(OAc)_2_, P­(^*t*^Bu)_3_BF_4_, ^t^BuONa, Toluene, 110 °C, 16 h, **90%**, (e); I_2_, MeOH 0 °C, 20 min, **87%** (f); Azetidine, Pd­(OAc)_2_, P­(^*t*^Bu)_3_. BF_4_, ^*t*^BuONa, Toluene, 110 °C, 16 h **66%**, (g); I_2_, MeOH, 30 min, 0 °C, 15 min, **90%**

### Photophysical Characterization

First, the absorption and emission spectra of both PSs, **NSeMorph** and **NSeAze**, were recorded in PBS buffer (pH 7.4, 1% DMSO). Both compounds exhibited absorption maxima at 671 nm (Figure 1a). The combined two-atom modifications on the rhodamine core, in fact, resulted in a perfect additive nature, and a ∼120 nm shift was observed. Achieving this shift while utilizing a heavy atom (selenium) in the core then resulted in molecules with low molecular weights, absorption in the NIR region, and phototoxic potential. Fluorescence spectra were obtained for **NSeMorph** and **NSeAze**, with emission maxima observed at 717 and 695 nm, respectively. The fluorescence quantum yield (Φ_F_) of **NSeMorph** was determined as 1.1%. However, as will be demonstrated, **NSeMorph** is sufficiently fluorescent to produce high-quality images by confocal microscopy. The impact of incorporating a four-membered azetidine ring into the classical xanthene core was first demonstrated by the Lavis group in 2015.^72^ The resulting Janelia Fluor (JF) dyes exhibited enhanced brightness and photostability, which were attributed to the suppression of twisted intramolecular charge transfer (TICT) state formation. The effect was apparent with selenium-containing cores as well, and a significant increase in fluorescence emission was observed for **NSeAze** with a Φ_F_ of 8.2% in methanol (methylene blue as a reference standard, Φ_F_ = 0.23 in MeOH) (Figure 1b and Table 1).

**Figure 1:**
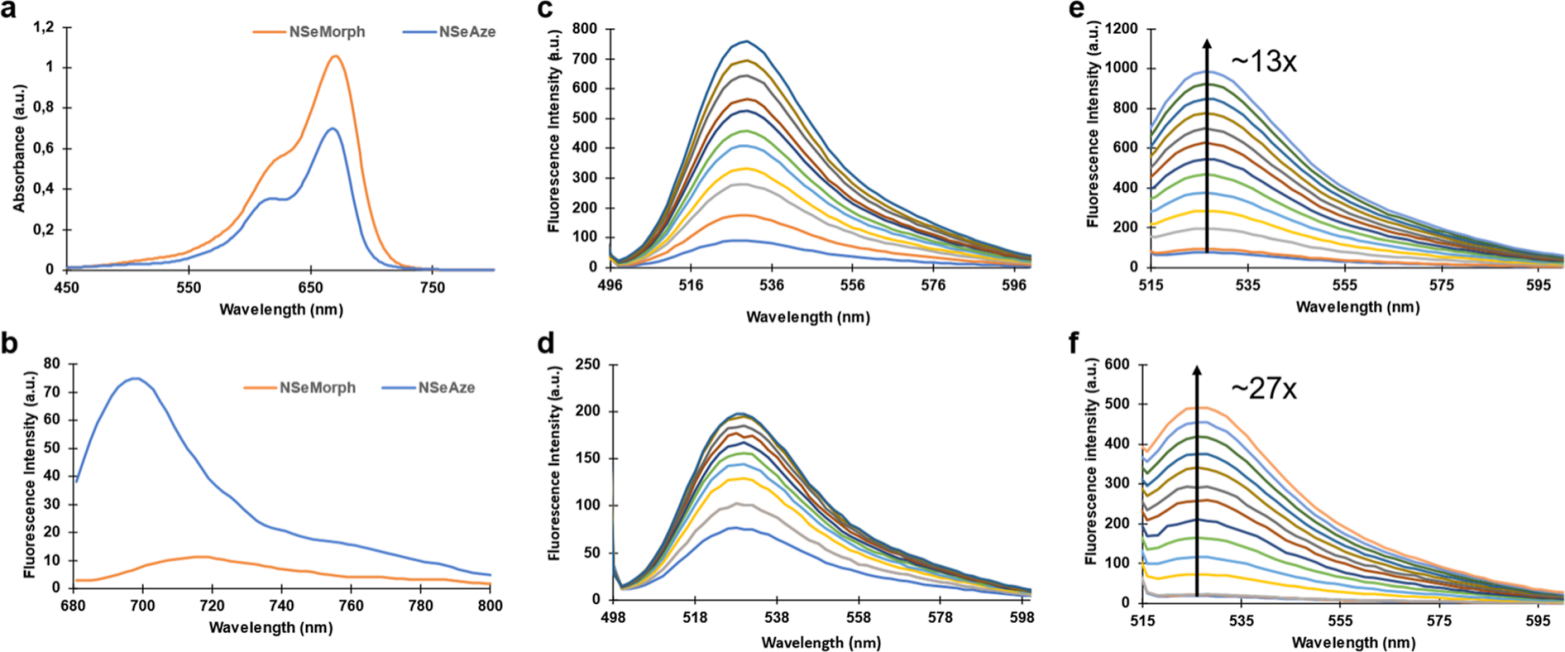
(a) Absorption spectrum of **NSeAze** (blue) and **NSeMorph** (orange) (20 μM) in PBS buffer (pH 7.4, 1% DMSO). (b) Emission spectrum of **NSeAze** (blue) and **NSeMorph** (orange) (40 μM) in PBS buffer (pH 7.4, 1% DMSO). Fluorescence spectra of SOSG (1 μM) containing (c) **NSeMorph** (20 μM) (d) **NSeAze** (20 μM) and fluorescence spectra of DHR 123 (1 μM) containing (e) **NSeMorph** (20 μM) and (f) **NSeAze** (20 μM) in PBS buffer (pH: 7.4, 1% DMSO) after LED light exposure (660 nm, 100 s total irradiation time) to detect ^1^O_2_ generation efficiency (c,d) and to detect O_2_^•–^ generation efficiency (e,f).

**Table 1:** Photophysical Properties of **NSeMorph** and **NSeAze**

PS	λ_abs_ (nm)^a^	λ_ems_ (nm)^a^	ε (M^–1^ cm^–1^)^a^	φ_F_ (%)^b^	φ_Δ_ (%)^c^	Log*P* ^d^
**NSeMorph**	671	717	16,600	1.1	75	–0.42
**NSeAze**	671	695	15,300	8.2	31	0.81

a In PBS (pH 7.4, 1% DMSO).

b Reference: methylene blue in MeOH (φ_F_ = 23%).

c Reference: methylene blue in PBS (Φ_Δ_ = 52%).

d Distribution coefficient in *n*-octanol/PBS solution.

Before singlet oxygen trapping experiments were conducted, the photostability of **NSeMorph** and **NSeAze** was evaluated under irradiation with a 660 nm LED light source (24.3 mW/cm^2^). Absorbance spectra were recorded after 10 s of light exposure at various pH values (5.4, 6.4, 7.4, and 8.4, Figure S5,6), and both **NSeMorph** and **NSeAze** showed no significant photodecomposition after 100 s of illumination. Subsequently, their singlet oxygen generation capabilities were assessed in PBS buffer. 2,2′-(Anthracene-9,10-diyl)­bis­(methylene)­dimalonic acid was used as a singlet oxygen trap to determine the quantum yields of singlet oxygen generation (Φ_Δ_). The calculated Φ_Δ_ values were 75% for **NSeMorph** and 31% for **NSeAze** in PBS buffer containing 1% DMSO, using methylene blue as a reference (Φ_Δ_ = 0.52 in PBS buffer). Notably, **NSeMorph** exhibited one of the highest singlet oxygen quantum yields reported in aqueous media (Figure S4 and Table 1). Furthermore, the ROS generation capabilities of the PSs were also evaluated using singlet oxygen sensor green (SOSG). Both **NSeMorph** and **NSeAze** were found to produce ^1^O_2_ upon light irradiation (Figure 1c,d).

Notably, **NSeMorph** induced a significantly greater increase in the fluorescence intensity of SOSG compared to **NSeAze**, further supporting the higher singlet oxygen quantum yield of **NSeMorph** (75%) relative to **NSeAze** (31%). Additionally, the type I ROS generation abilities of the PSs were assessed in PBS (pH 7.4) using dihydrorhodamine 123 (DHR123). In addition to the type II mechanism, both PSs also demonstrated a substantial increase in the emission of oxidized DHR123, supporting ROS generation via the type I pathway (Figure 1e,f). It is important to note that **NSeAze** showed a 27-fold increase upon irradiation, compared to a 13-fold increase for **NSeMorph**.

### Cytotoxicity Analysis

The time- and concentration-dependent effects of **NSeAze** and **NSeMorph** on glioblastoma cells were determined by using the MTT assay (Figure S7). In this regard, U118MG and U87MG cells were administered with **NSeMorph** (0.5–20 μM) and **NSeAze** (0.01–2.5 μM) for 24 h to evaluate their dark toxicity. Before PDT application, both cell lines were treated as indicated for 1 h and then exposed to 660 nm LED light for 2 and 1 h, respectively (Figure 2). Following overnight incubation, the resulting cell viability was calculated and compared to the effect of both compounds in the absence of irradiation. The IC_50_ values for **NSeMorph** were determined as 15.85 ± 0.84 and 8.02 ± 0.34 μM in U87MG and U118MG cell lines, respectively. In contrast, **NSeAze** exerted a significantly enhanced cytotoxic effect with IC_50_ values of 456 ± 21 nM for U87MG and 461 ± 19 nM for U118MG cells. The photodynamic efficacy and selectivity of **NSeMorph** and **NSeAze** were quantitatively assessed using various established indices, including PI,^85,86^ selectivity index (SI),^87,88^ and the in vitro therapeutic index (TI) (Table 2).^89,90^

**Figure 2:**
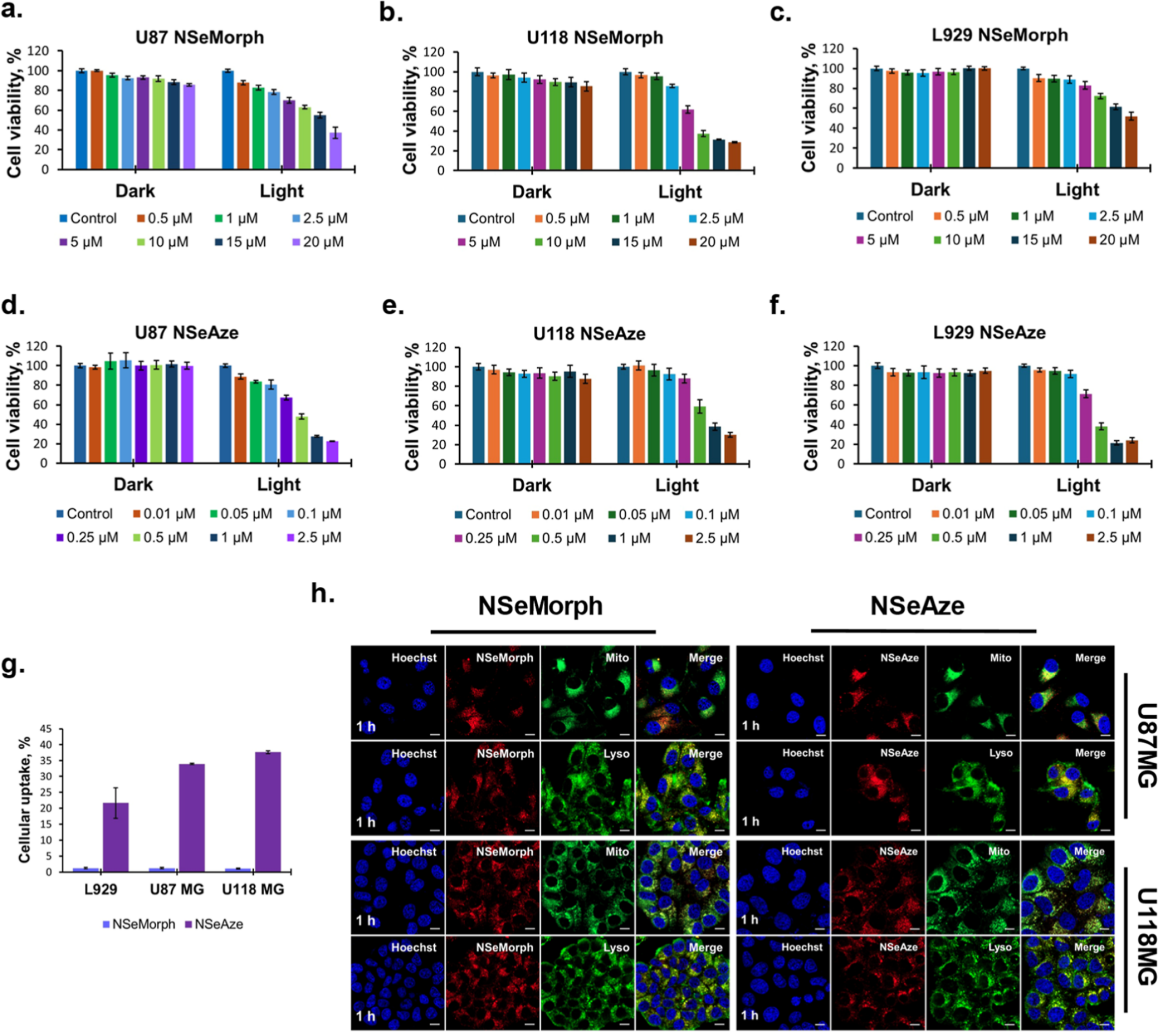
Cell viability rates in **NSeMorph**- and **NSeAze**-treated U87MG (a,d), U118MG (b,e) glioblastoma cells and L929 (c,f) healthy cells in the presence or absence of irradiation (dark and light). Cells were treated with both compounds at varying concentrations and incubated for either 24 h for dark toxicity or 1 h for PDT application. Following the treatment, cells were exposed to LED light for 2 h in **NSeMorph**-treated cells and for 1 h in **NSeAze**-treated groups. The resulting viability was plotted against the untreated control. Quantification of cellular uptake (%) of **NSeMorph** and **NSeAze** (100 μM, 1 h) in L929, U87MG, and U118MG cells (g). Subcellular colocalization of **NSeMorph** (2.5 μM) and **NSeAze** (2.5 μM) in U87MG and U118MG cells, visualized by confocal microscopy (h). Blue: Hoechst 33342 (nuclei); red: **NSeMorph** or **NSeAze**; green: MitoTracker Green FM or LysoTracker Yellow HCK-123. Scale bar: 10 μm.

**Table 2:** Comparison of Phototoxicity Characteristics of **NSeMorph** and **NSeAze**

Cell line	PS	IC_50_ (dark, μM)	IC_50_ (light, μM)	PI^a^	SI^b^	TI^c^
U87MG	**NSeMorph**	>500	15.90 ± 0.84	>31	>1.26	>32
	**NSeAze**	65.9 ± 2.5	0.46 ± 0.05	145	0.99	288
U118MG	**NSeMorph**	374.8 ± 17.4	8.02 ± 0.34	47	>2.49	>62
	**NSeAze**	60.4 ± 2.6	0.46 ± 0.02	131	0.98	281
L929	**NSeMorph**	>500	>20	NA	NA	NA
	**NSeAze**	129.4 ± 5.1	<1	NA	NA	NA

a Phototoxicity index = IC_50,dark_/IC_50,light_.

b SI = IC_50,light,healthycells_/IC_50,light,cancercells._

c *In vitro* TI = IC_50,dark,healthycells_/IC_50,light,cancercells_.

**NSeAze** demonstrated high photodynamic potency in U87MG and U118MG cells, with PI values of 144.7 and 131.0, respectively. However, **NSeAze** also exhibited measurable dark toxicity, with IC_50_ values of 65.85 ± 2.51 μM (U87MG), 60.4 ± 2.65 μM (U118MG), and 129.4 ± 5.06 μM in healthy L929 cells, suggesting a narrower therapeutic window compared to **NSeMorph**. In contrast, **NSeMorph** displayed superior dark safety, with no measurable cytotoxicity up to 500 μM in U87MG and L929 cells, and only moderate dark toxicity in U118MG (IC_50_ = 374.8 ± 17.4 μM) (Figure S8). According to the light-activated condition, PI values of **NSeMorph** in U87MG and U118MG were calculated as >31 and 46.7, respectively. Notably, the *in vitro* TI was highest for **NSeAze** in U87MG (TI = 287.6) and U118MG (TI = 281.1), though this was partly due to its low phototoxic IC_50_. **NSeMorph** demonstrated a more substantial dark safety margin, particularly in L929 cells (TI > 62.3 for U118MG). SI values reflected comparable tumor-to-normal distinction under light conditions (0.98–0.99 for **NSeAze**, >1.26–>2.49 for **NSeMorph**) (Figures S7 and S8 and Table 2). Together, these findings suggest that while **NSeAze** possesses higher intrinsic phototoxic efficacy, **NSeMorph** may offer a safer phototherapeutic profile due to its minimal dark toxicity (Figure 2a–f).

To elucidate the mechanism underlying the PDT efficacy of both compounds, a series of scavengers (*N*-acetylcysteine (NAC), trolox, sodium azide (NaN_3_), histidine, mannitol, and tiron) was administered to treated cells (at IC_50_ of **NSeMorph** and **NSeAze**) 1 h before PDT application, and the cell viability was determined following overnight recovery. Results indicated that NAC and NaN_3_ prevent cell death significantly, suggesting the involvement of both type I and type II ROS. Additionally, histidine (OH^**.**^ and superoxide scavenger) also demonstrated the ability to reduce the effects of both agents in both cell lines (particularly in U87MG), supporting type I ROS involvement. However, the other scavengers, trolox, tiron, and mannitol, exhibited diverse inhibitory effects compared to those observed with NAC and NaN_3_, confirming the presence of other types of free radicals, which nonetheless do not appear to be predominant (Figure 3a,b).

**Figure 3:**
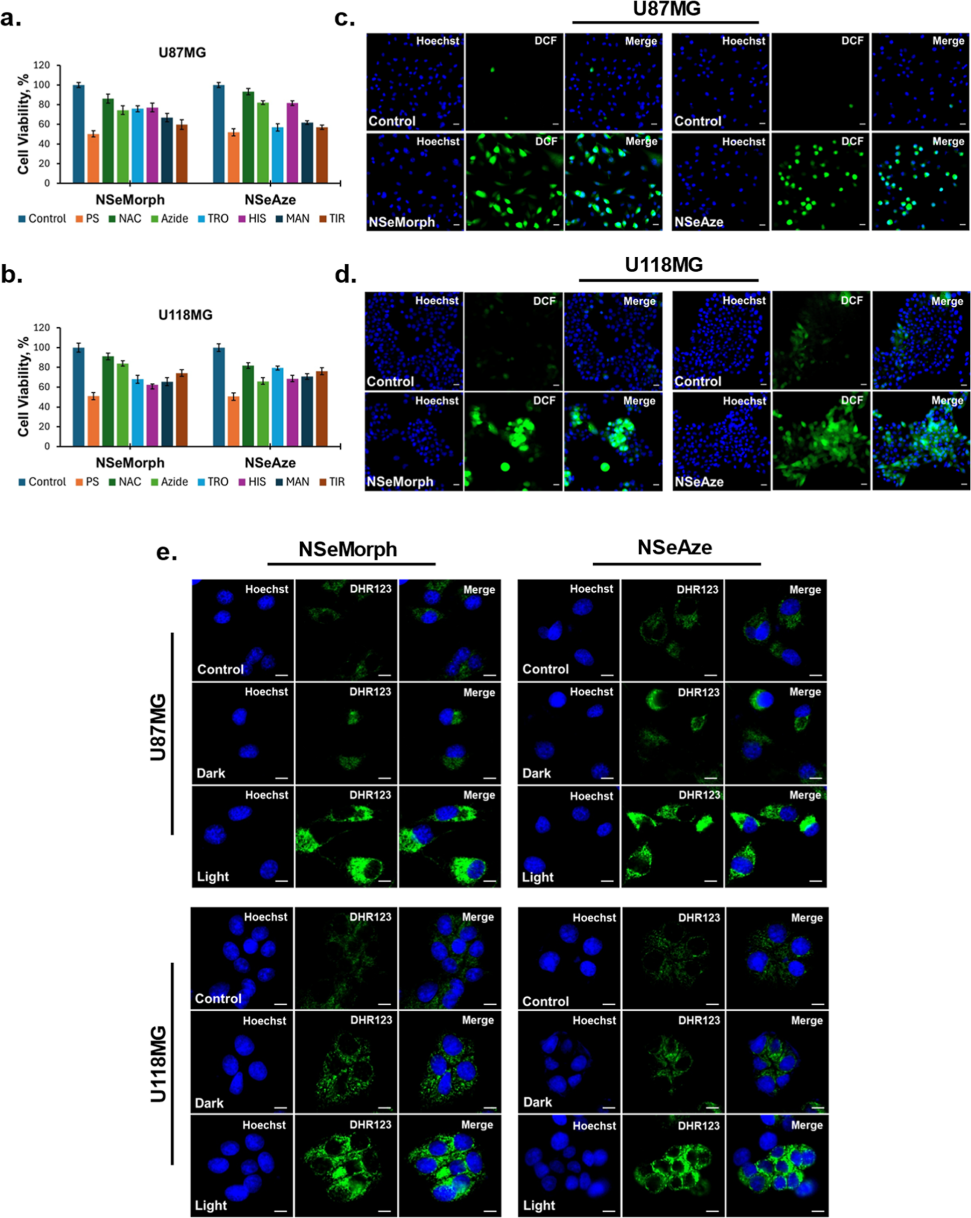
Effect of **NSeMorph** and **NSeAze** (IC_50_ values) in U87MG and U118MG glioblastoma cells in the presence or absence of scavengers (a,b). ROS detection with DCFH-DA staining in U87MG and U118MG cells exposed to irradiation (**1 or 2 h**) following the treatment (c,d). Scale bar: 20 μm. Detection of superoxide anion with DHR123 (5 μM) staining in U87MG and U118MG cells treated with the IC_50_ values of **NSeMorph** and **NSeAze** under dark and light conditions (e). Scale bar: 10 μm.

### Cellular Uptake and Internalization

The cellular uptake efficiency of **NSeAze** and **NSeMorph** was quantitatively assessed in glioblastoma cell lines (U87MG and U118MG) and a nonmalignant fibroblast line (L929). **NSeAze** demonstrated significantly higher internalization across all tested cell types compared to **NSeMorph**, with a clear tumor selectivity profile. In both U87MG and U118MG cells, **NSeAze** uptake exceeded 34–38%, whereas in L929 cells, it remained lower (∼22%), indicating preferential accumulation in glioma cells. In contrast, **NSeMorph** uptake was consistently minimal (<2%) across all lines, suggesting a low-affinity or inefficient internalization mechanism along with a lower rate of cell death (Figure 2g). We believe that the drastic difference in cellular uptake is primarily related to the significant difference in the partition coefficients of the two agents (Table 1). It is remarkable that even though **NSeMorph** has four additional CH_2_ moieties compared to **NSeAze**, the presence of additional oxygens in **NSeMorph** resulted in significantly higher water solubility, presumably via acting as H-bond acceptors.

To understand the dynamics of photosensitizer uptake, time-dependent confocal microscopy was performed using Hoechst 33342 counterstaining. Both compounds were evaluated at 2.5 μM in U87MG and U118MG cells across 0.5, 1, 2, and 4 h post-treatment (Figure S9). **NSeAze** demonstrated rapid perinuclear accumulation with diffuse distribution as early as 30 min, intensifying gradually over time. The fluorescence pattern was homogeneous, suggesting passive diffusion followed by potential retention in cytosolic or organelle compartments. Its fluorescence emission was consistently higher than that of **NSeMorph** at all time points, supporting the quantitative uptake trends observed previously. **NSeMorph**, in contrast, showed delayed and punctuated intracellular fluorescence, with noticeable emission detected only after 1–2 h. The granulated pattern may imply possible sequestration in vesicular structures.

### Subcellular Co-Localization

Subcellular localization is a key factor that determines the therapeutic outcome in PDT.^91^ Targeted localization is the main goal in these applications since elucidating the site of accumulation within the cell provides insight into the cell death mechanism triggered by PDT agents. Furthermore, mitochondria are the primary site of energy production, which also generates ROS; hence, a mitochondria-targeted approach increase the efficacy of PS agents.^92,93^ Additionally, mitochondrial or lysosomal localization is often an advantage in cancer treatment since it suggests the induction of controlled cell death rather than uncontrolled necrosis.^94^

Following the cellular uptake analysis, the intracellular trafficking dynamics and suborganelle targeting of **NSeAze** and **NSeMorph** were investigated via time-dependent live-cell confocal microscopy in U87MG and U118MG glioblastoma cells to visualize lysosomal and mitochondrial compartments (Figure 2h). Both compounds were administered at a 2.5 μM concentration and visualized at 0.5, 1, 2, and 4 h postincubation (Figures S10 and S11). Co-localization patterns were quantified using Pearson correlation coefficients (PCC), derived from fluorescence intensity scatterplots between the red channel (PS) and respective green organelle markers. Confocal images demonstrated that **NSeMorph** exhibits a dynamic redistribution profile in U87MG and U118MG cells. In the early phase (0.5–1 h), partial colocalization with mitochondria was noticeable (PCC = 0.78 and 0.81 in U87MG and U118MG, respectively). Still, this association declined over time (PCC at 4 h = 0.69 in U118MG), suggesting transient mitochondrial localization. In contrast, lysosomal colocalization was more stable and consistent, with PCC values ranging from 0.75 to 0.82 across time points, indicating a preferential lysosomal localization. These findings are potential indicators of lysosomotropic accumulation, a feature commonly associated with **NSeMorph** internalization via endocytosis, followed by endosomal maturation. **NSeAze**, however, displayed a more persistent mitochondrial association. Co-localization with mitochondria increased rapidly, reaching peak PCC values of 0.84–0.89 at 1–2 h in both cell lines, and remained elevated thereafter. Lysosomal colocalization was also detectable (PCC ∼ 0.79–0.84), but clearly secondary to mitochondrial enrichment.

The gradual but pronounced lysosomal accumulation over time of **NSeMoprh**, coupled with only moderate mitochondrial targeting, restrict its ability to induce cell death via direct mitochondrial ROS production effectively. Conversely, **NSeAze** achieves rapid and efficient mitochondrial localization within the first hour of treatment in addition to significant lysosomal localization as well. This dual-phase distribution, characterized by early mitochondrial presence followed by delayed lysosomal capture, may have enabled **NSeAze** to exert a phototoxic effect via both mitochondrial oxidative stress and possibly lysosomal disruption. To eliminate the possibility of degradation in lysosomes due to their acidic nature, the stabilities of both **NSeMorph** and **NSeAze** were evaluated at different pHs under both dark and light conditions. The results demonstrated that both agents remain stable under all conditions (Figures S5 and S6).

Further analysis was conducted to elucidate mitochondrial- or lysosomal-localization-dependent cell death mechanisms. For this purpose, lipid peroxidation, a major end product of ROS-mediated cellular damage, was assessed.^95^ The results demonstrated that **NSeAze** led to a robust increase in lipid peroxidation immediately following light exposure, which further escalated during the postirradiation resting period and remained elevated for up to 24 h in both cell lines, with 245.24 ± 5.92% in U118MG, 309.64 ± 13.20% in U87MG cells. **NSeMorph** also induced a significant increase in lipid peroxidation (244.22 ± 2.38% in U118MG; 216.73 ± 3.98% in U87MG); however, this effect required substantially higher concentrations (10–15 μM) compared to **NSeAze**. Moreover, the pronounced rise in lipid peroxidation was predominantly evident during the 24 h resting stage rather than immediately after light exposure, as observed with **NSeAze**. The apparent increase in lipid peroxidation explains the damage to the membranous structures of cells, such as mitochondria, lysosomes, and plasma membranes (Figure 4a,b). This was further confirmed by the detection of free unsaturated lipid content of cells, which is released upon disruption of the membranous structures. In this regard, the sulfo-phosphovanillin assay indicated that both compounds induce a significant release of lipid products (Figure 4c,d), with a particularly notable increase in **NSeAze**-treated cells.

**Figure 4:**
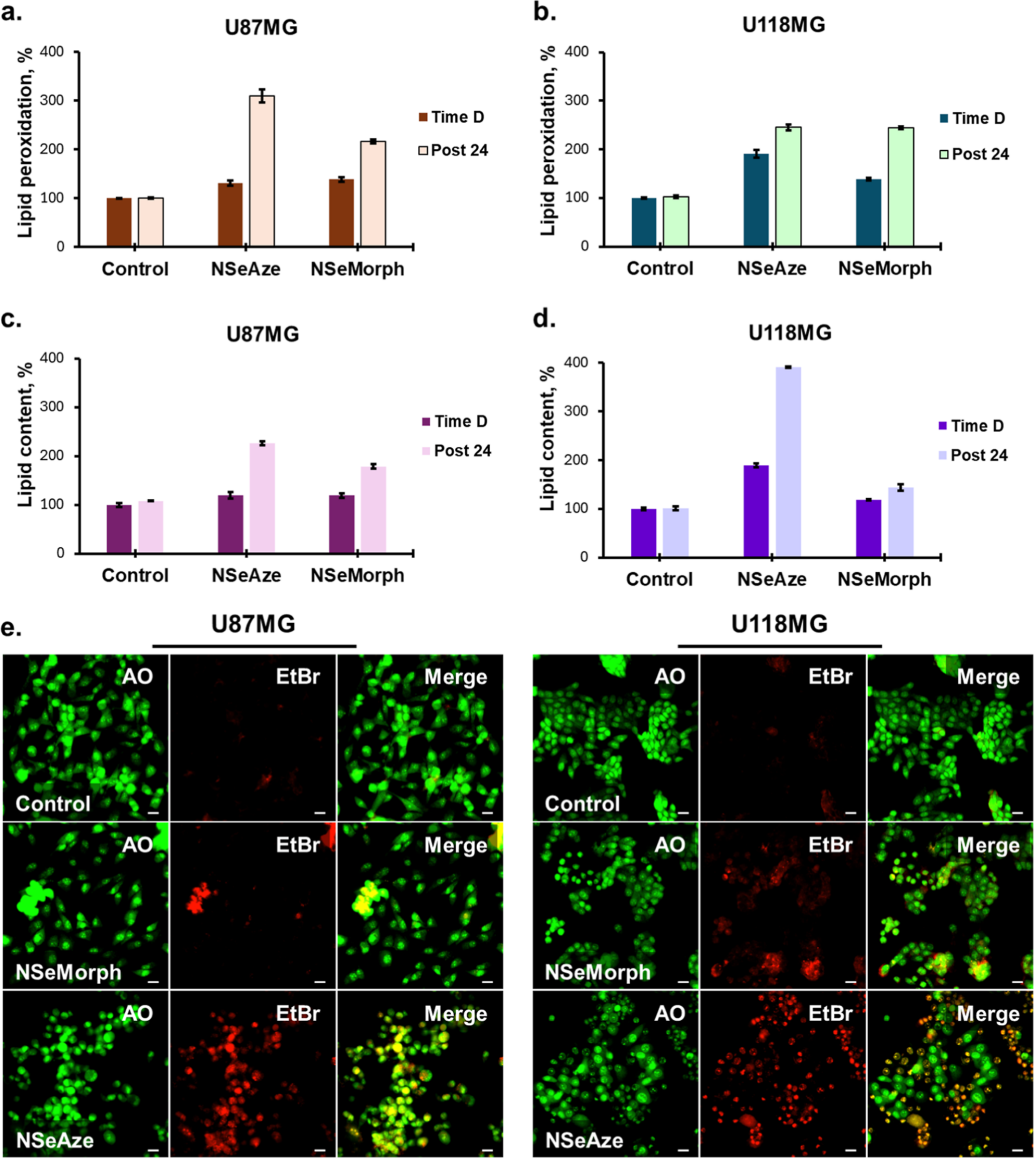
Lipid peroxidation percentages measured immediately after irradiation (time D) and 24 h postirradiation (post 24) in **NSeMorph-** or **NSeAz-**treated U87MG (a) and U118MG (b) cells (*n* = 6). Cellular lipid content was determined at time D and Post 24 in **NSeMorph-** or **NSeAze**-treated U87MG (c) and U118MG (d) cells (*n* = 6). Representative confocal images showing acridine orange (AO, green) and ethidium bromide (EtBr, red) dual staining in **NSeMorph**- and **NSeAze**-treated U87MG and U118MG cells upon irradiation (e). Merged images represent live (green) and apoptotic/necrotic (orange-red) populations (*n* = 6). Scale bar: 10 μm.

In summary, **NSeAze** and **NSeMorph** both undergo time-dependent internalization into lysosomal compartments but differ substantially in their mitochondrial colocalization and temporal activation potential. **NSeAze** demonstrates a more favorable intracellular distribution profile for photodynamic therapy, characterized by rapid mitochondrial targeting and early ROS-mediated cytotoxicity, whereas **NSeMorph**’s predominant lysosomal localization, slower accumulation, and release profile limit its cytotoxic activity (Figures S10 and S11). In addition, **NSeAze**’s ability to induce higher levels of lipid peroxidation and concomitant release of lipid components from crucial organelles supports the more substantial phototoxic effect of **NSeAze** starting from the early stages of treatment and persisting up to 24 h.

### Cell Death Mechanism

The rapid mitochondrial disruption often shifts the balance toward irreversible cell death pathways.^96^ Consistent with the above-mentioned mechanistic evidence, the cell death analysis demonstrated clear differences in the cytotoxic profiles of **NSeAze** and **NSeMorph**. In this regard, acridine orange/ethidium bromide (AO/EtBr) staining was performed to detect cell death. This staining method relies on the differential uptake and fluorescence of AO, which permeates all cells and emits green fluorescence in viable cells but shifts to a yellowish hue in early apoptotic cells due to chromatin condensation, whereas EtBr selectively enters cells with compromised membranes and exhibits red fluorescence, thereby marking late apoptotic/necrotic cell populations.^97^ The confocal images obtained upon staining revealed that in U87MG cells both compounds triggered early apoptosis, but the effect was more pronounced in **NSeAze**-treated groups. In U118MG cells, **NSeMorph** induced a similar early apoptotic pattern, whereas **NSeAze** treatment led predominantly to late apoptosis/necrosis (Figure 4e). These findings confirm the rapid-onset and sustained phototoxic profile of **NSeAze**, whereas **NSeMorph** exhibited a delayed-onset response.

### ROS Detection and Profiling

Cancer cells generally exhibit altered redox homeostasis with increased oxidative stress due to their high metabolic activity. The elevation of the ROS promotes genomic instability and uncontrolled proliferation. However, excessive ROS generation may reverse the situation, leading to irreversible cellular damage and death.^98,99^ Therefore, augmentation of oxidative stress has become a prominent target in cancer treatment strategies.^100^ The use of photosensitizers is a promising approach in this regard, which produces a significant amount of ROS. Numerous studies reported the potential of PDT agents in cancer research due to the significant cytotoxicity obtained through increased oxidative stress.^92^

The intracellular ROS-inducing potential of **NSeAze** and **NSeMorph** was investigated through live-cell confocal imaging using DCFH–DA and DHR123 probes (Figures 3c–e), in combination with ROS scavengers, to elucidate the dominant reactive species involved. U87MG and U118MG glioblastoma cells were treated with IC_50_ concentrations of each compound and subjected to photoirradiation in the presence or absence of scavengers (Figures S12 and S13). In DCFH–DA–based assays, which primarily detect general ROS, both photosensitizers produced a robust green fluorescence signal upon photoactivation, indicating significant intracellular ROS generation. Scavenger cotreatment provided key mechanistic insights into the type and origin of the ROS species. The singlet oxygen (^1^O_2_) quencher NaN_3_ substantially reduced DCF fluorescence in both **NSeAze**-treated cell lines, with a more complete suppression observed in U87MG cells than in U118MG cells. Similarly, NAC, a thiol-based ROS quencher, led to a pronounced reduction in fluorescence intensity in both cell lines, confirming the contribution of peroxyl and hydroperoxide radicals to the total ROS pool. This observation strongly suggests that **NSeAze** is capable of generating a mixed profile of ROS (type I and type II) in both cell lines with a predominance of type II radicals that appears to be cell-type dependent. Furthermore, histidine, which neutralizes singlet oxygen and partially scavenges hydroxyl radicals, also reduced the DCF signal in U87MG cells, albeit to a lesser extent than in NaN_3_. In contrast, mannitol, a hydroxyl radical scavenger, and tiron, a superoxide and iron-chelating antioxidant, had only slight suppressive effects in both cell lines, indicating that hydroxyl and superoxide radicals are not the predominant contributors in the DCF-detected ROS population. Similar results were obtained in **NSeMorph**-treated cells, where NAC and NaN_3_ exhibited the most potent suppression of the fluorescence. However, unlike **NSeAze**-treated cells, both U87MG and U118MG cell lines demonstrated similar extents of inhibition.

In order to specifically examine mitochondrial oxidative stress, live-cell imaging with DHR123 was conducted (Figures S14 and S15). DHR123 becomes fluorescent upon oxidation by superoxide within mitochondria, offering compartment-specific insight into type I photodynamic effects. Both compounds induced clear DHR123 fluorescence upon light exposure. Significantly, this signal was dramatically attenuated by NAC and NaN_3_ in both cell lines, confirming the involvement of mitochondrial superoxide. Trolox also reduced the DHR123 fluorescence in **NSeMorph**-treated cells, supporting the presence of peroxyl and other redox-active species. On the other hand, histidine, mannitol, and tiron had minimal impact on DHR123 intensity, which is consistent with DCF assay results.

The differential scavenger inhibition profiles strongly support a mixed photodynamic mechanism, with **NSeMorph** exhibiting a dominant type II ROS signature and secondary mitochondrial oxidative contributions, whereas **NSeAze** favors a varying ROS profile with a relatively greater reliance on type I mitochondrial superoxide production in U118MG cells and type II in U87MG cells. These conclusions are further corroborated by cell-free photochemical validation, in which SOSG and DHR fluorescence spectra confirmed both compounds’ ability to generate ROS upon irradiation.

## Conclusion

In summary, this work demonstrates the design, synthesis, and photodynamic efficacy of two novel selenium-containing xanthane-based photosensitizers, **NSeMorph** and **NSeAze**, tailored explicitly for glioblastoma treatment. By achieving absorption above 650 nm while keeping the molecular weights below 420 g/mol, these compounds address a long-standing limitation in PDT for brain cancers: the trade-off between near-infrared activation and blood-brain barrier permeability. Both compounds exhibited significant phototoxicity against glioblastoma cell lines (U87 and U118), yet their selectivity and potency varied markedly, depending on the nature of the donor side group.

**NSeMorph**, containing morpholine donors, displayed moderate cytotoxicity under light activation with notable selectivity against cancer cells over healthy cells (L929), despite the absence of targeting ligands or activatable cage moieties. This observation suggests that donor-group-induced physicochemical changes may enhance passive selectivity, potentially through differences in uptake or subcellular localization. In contrast, **NSeAze**, bearing azetidine donors, exhibited dramatically improved potency, with IC_50_ values of 456 nM for the U87MG cell line and 461 nM for the U118MG cell line. To the best of our knowledge, these are the lowest IC_50_ values reported in the literature against glioblastoma (U87MG and U118MG) that combine NIR absorption in aqueous media and low molecular weight (<400 g/mol). Its high phototoxicity index further supports its potential for potent light-induced cytotoxic action. Detailed *in vitro* studies revealed small structural changes resulted in significantly different organelle localization and retention, which then contributed notably to PDT efficacy and selectivity.

These findings emphasize that subtle structural variations, such as changes in donor side groups, can profoundly influence both the efficacy and the selectivity of xanthane-based PDT agents. To the best of our knowledge, this is one of the first studies to demonstrate such a pronounced donor-dependent modulation of PDT activity in brain cancer models. These insights not only provide valuable structure–activity relationship information for xanthene-based chromophores but also open up new directions for developing more selective, brain-penetrating photosensitizers for challenging cancers such as glioblastoma.

## Supplementary Material



## Notes

Synthetic details, NMR spectra, and additional photophysical/*in vitro* characterizations (PDF)
