# Supplementary material for: Drastic Impact of Donor Substituents on Xanthenes in the PDT of Glioblastoma
Source: JACS Au. 2025 Oct 1;5(11):5346–58. doi: 10.1021/jacsau.5c00738 (PMC12648288; doi:10.1021/jacsau.5c00738)
Supplement: Supplementary file 1 [file au5c00738_si_001.pdf]

Supporting Information for

Drastic Impact of Donor Substituents on Xanthenes in the PDT of Glioblastoma

O. Karaman[a,†], E. Yesilcimen[a,†], M. Forough[a], Z. Elmazoglu[a,b,*], G. Gunbas[a,*]

[a] Department of Chemistry, Middle East Technical University, 06800, Ankara, Turkiye
[b] Faculty of Pharmacy, Ankara Medipol University, 06050, Ankara, Turkiye
[†]Contributed equally
* Prof. Dr. Gorkem Gunbas, ggunbas@metu.edu.tr
* Assist. Prof. Dr. Zubeyir Elmazoglu, zubeyir.elmazoglu@ankaramedipol.edu.tr

Contents

General: 2
Synthesis 3
Synthesis of compound **2** 3
Synthesis of compound **3** 4
Synthesis of compound **4** 4
Synthesis of compound **5** 5
Synthesis of compound **6** 5
Synthesis of **NSeMorph** 6
Synthesis of **NSeAze** 6
HPLC Analysis 7
Photophysical Characterization 8
Fluorescence Quantum Yield 8
Singlet Oxygen Trap Experiment 9
SOSG Oxidation in the Presence of $^1O_2$ 13
DHR Oxidation for Detection of Superoxide anion O_2 13
Cell Culture and Treatment 13
Cell Viability Analysis 14
Cellular Uptake Assay 16
Confocal Imaging Studies 17
Cellular internalization 17
Subcellular co-localization 19
Reactive oxygen species (ROS) detection 20
Mitochondrial superoxide detection 24
Determination of lipid peroxidation 27
Sulfo-phospho-vanillin assay 27
Acridine Orange/Ethidium Bromide (AO/EtBr) staining for cell death 27
NMR Spectra 30
HRMS Results 40
References 41

General:

The starting materials and solvents were purchased from Sigma Aldrich, abcr, TCI, and Merck. Solvents used for column chromatography, Hexane, EtOAc, and DCM, were distilled prior to use over $CaCl_2$. All reactions were conducted under a nitrogen atmosphere, unless otherwise specified. Reaction solvents (Diethyl ether, THF, DCM, Toluene, and DMF) were directly used from MBraun MBSPS5 solvent drying system. The reactions were monitored by thin-layer chromatography (TLC) (Merck Silica Gel 60 F254) and visualized by UV light at 245 nm and 366 nm. Column chromatography of all products was performed using Merck Silica Gel (particle size: 0.040-0.063 mm, 230-400 mesh ASTM).

The structural analysis of the synthesized compounds was conducted using NMR and HRMS. ^{1}H and ^{13}C nuclear magnetic resonance spectra of the compounds were recorded in deuterated solvents with Bruker Avance III Ultrashield 400 Hz NMR spectrometer. The chemical shifts were stated in parts per million (ppm) with tetramethylsilane (TMS) as an internal reference. High-Resolution Mass Spectra (HRMS) were processed for the novel compounds using Time of Flight mass analyzer with Water Synapt MS System.

The photophysical analyses were performed through absorbance and fluorescence measurements. Absorption spectra were collected using a double beam Jasco V-730 (Easton, MD, USA) UV-vis spectrophotometer. A Cary Eclipse fluorescence spectrophotometer (Santa Clara, CA, USA) equipped with a Cary single cell peltier (Agilent Technologies) temperature controller was used to record the fluorescence emission spectra. All measurements were performed at room temperature using 10 mm quartz (3.5 mL, 111-QS, Hellma).

A custom-made LED array was used as the light source with an emission maximum at 660 nm and power density of 24.3 mW/cm^2.

Synthesis

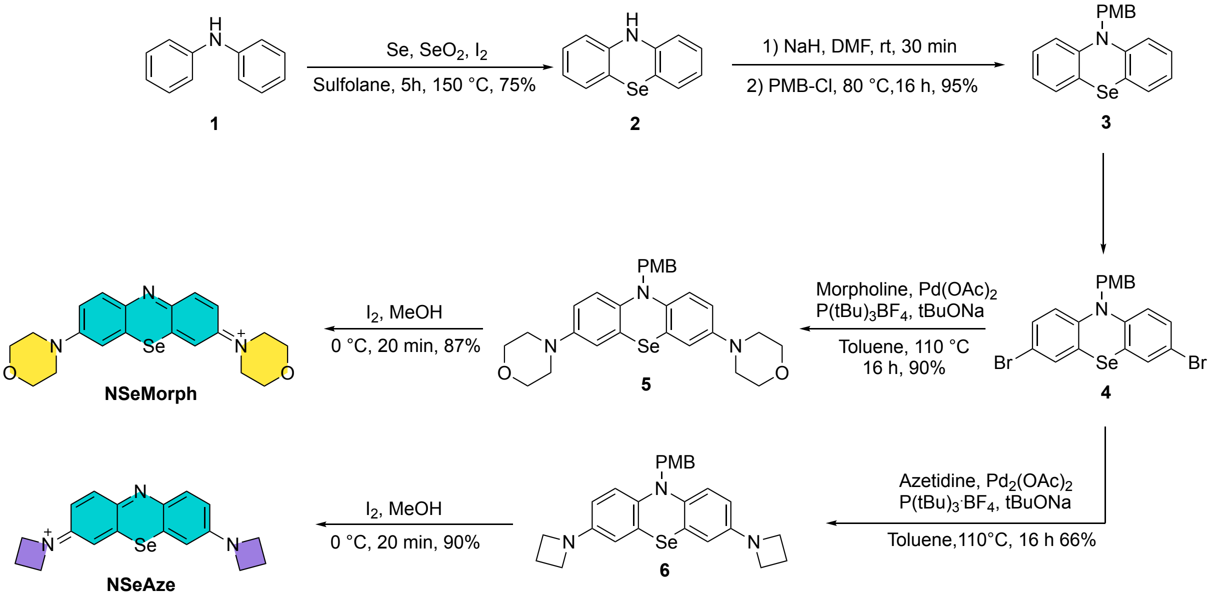

Scheme S1. Synthetic pathway for **NSeMorph** and **NSeAze**

Synthesis of compound **2**

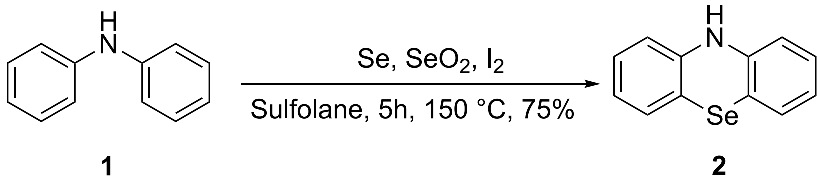

To a 25 mL Schlenk tube, diphenylamine (4.32 g, 25.58 mmol, 2 eq.) was added, and vacuum–N_2 was applied. Then, selenium powder (1.00 g, 12.79 mmol, 1 eq.), SeO_2 (1.70 g, 15.35 mmol, 1.2 eq.), and I_2 (162.6 g, 1.28 mmol, 0.1 eq.) were added and dissolved in sulfolane (5 mL). The reaction mixture was heated to 150 °C and stirred at that temperature for 16 hours. After the conclusion of the reaction, the mixture was cooled to room temperature and filtered through celite, then washed with $CHCl_3$. The crude product was purified with column chromatography (silica, hexane:EtOAc, 5:1), and the target product, compound **2**, was obtained as an off-white solid (2.54 g, 75% yield). ^{1}H NMR (400 MHz, DMSO) δ 8.62 (s, 1H), 7.09 (d, J = 7.4 Hz, 2H), 7.03 (t, J = 7.6 Hz, 2H), 6.77 (d, J = 8.4 Hz, 4H). ^{13}C NMR (100 MHz, DMSO) δ 142.2, 128.9, 127.9, 122.2, 115.2, 111.6.

Synthesis of compound **3**

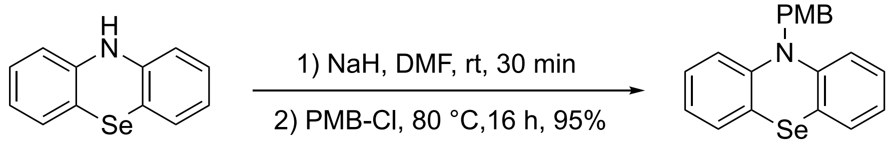

2 **3**

To a 50 mL Schlenk tube, compound **2** (1.27 g, 5.16 mmol) was added, and vacuum–N_2 was applied. Then, it was dissolved in dry DMF (20 mL) and cooled to 0°C. Afterward, NaH (0.41 g, 10.32 mmol, 2 eq., 60%) was added and warmed to room temperature, then stirred for 30 min at that temperature. Subsequently, PMB-Cl (1.61 g, 4.06 mmol, 2 eq.) was added, and the mixture was heated to 80 °C and stirred for 16 hours. After the completion of the reaction, the reaction mixture was cooled to room temperature, quenched with water, and extracted with Et_2O. The collected organic phases dried over $MgSO_4$, filtered and solvent evaporated. The crude product was purified by column chromatography (alumina, hexane:DCM, 2:1), yielding the target product, compound **3**, as a white solid (1.65 g, 95%). ^{1}H NMR (400 MHz, $CDCl_3$) δ 7.30 (dd, J = 12.7, 8.1 Hz, 4H), 7.08 (t, J = 7.7 Hz, 2H), 6.90 (t, J = 7.4 Hz, 2H), 6.84 (dd, J = 8.5, 2.5 Hz, 4H), 5.08 (s, 2H), 3.77 (s, 3H). ^{13}C NMR (100 MHz, $CDCl_3$) δ 158.4, 144.9, 129.5, 128.9, 128.1, 127.4, 123.0, 120.5, 117.0, 113.9, 55.1, 52.9. HRMS calculated for $C_{20}H_{17}NOSe$: 367.0475. Found: 367.0475. HRMS (ESI/MS) *m/z*: [M+H]+ Calcd. for $C_{20}H_{17}NOSe^+$ 367.0475; Found 367.0475.

Synthesis of compound **4**

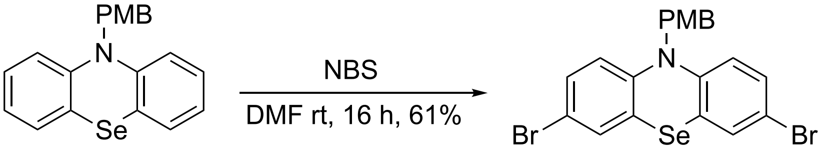

3 **4**

To a 50 mL two-necked round-bottom flask, compound **3** (0.61 g, 1.81 mmol) was added, and vacuum–N_2 was applied. Then, it was dissolved in dry DMF (20 mL). Subsequently, NBS (0.81 g, 4.53 mmol, 2.5 eq.) was added in portions over 1 hour. After the completion of the addition, the reaction mixture was stirred for 16 hours at room temperature. Then, the crude product was quenched with water and extracted with Et_2O. The collected organic phases dried over $MgSO_4$, filtered and solvent evaporated. The crude product was purified by column chromatography (alumina, hexane:DCM, 4:1), yielding the target product, compound **4**, as a white solid (0.58 g,

61%). ^{1}H NMR (400 MHz, $CDCl_3$) δ 7.38 (s, 2H), 7.22 (d, J = 8.1 Hz, 2H), 7.15 (d, J = 8.5 Hz, 2H), 6.84 (d, J = 8.0 Hz, 2H), 6.65 (d, J = 8.6 Hz, 2H), 4.95 (s, 2H), 3.77 (s, 3H). ^{13}C NMR (100 MHz, $CDCl_3$) δ 158.8, 143.9, 131.7, 130.4, 128.2, 127.9, 122.2, 118.4, 115.7, 114.2, 55.3, 53.2. HRMS calculated for $C_{20}H_{15}NOSeBr_2$: 524.8665. Found: 524.8665

Synthesis of compound **5**

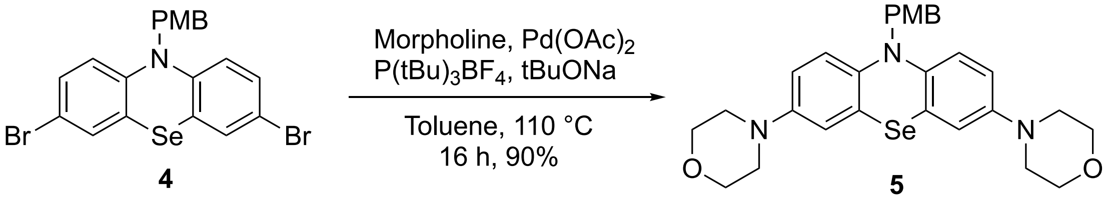

To a 25 mL Schlenk tube, compound **4** (0.17 g, 0.32 mmol), $Pd(OAc)_2$ (1.5 mg, 2%), $P(^tBu)_3BF_4$ (2.8 mg, 3%), Na^tBuO (0.09, 0.96 mmol, 2.95 eq.) and morpholine (0.10, 1.12 mmol, 3.5 eq.) were added, and vacuum–N_2 was applied. Then, toluene (5 mL) was added and degassed for 1 hour. After that, morpholine (0.09 g, 0.97 mmol, 3.5 eq.) was added and heated to 115 °C and stirred for 16 hours. After the completion of the reaction, the reaction mixture was cooled to room temperature and filtered through celite, then washed with $CHCl_3$. The crude product was purified with column chromatography (alumina, hexane:EtOAc, 10:1), and the target product, compound **5**, was obtained as an off-white solid (0.16 g) and used in next step without further purification.

Synthesis of compound **6**

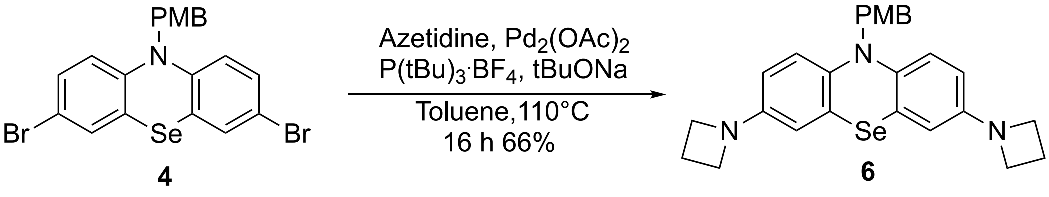

To a 25 mL Schlenk tube, compound **4** (0.2 g, 0.38 mmol), $Pd(OAc)_2$ (4.0 mg, 2%), $P(^tBu)_3BF_4$ (6.0 mg, 3%), Na^tBuO (0.11, 1.13 mmol, 2.95 eq.) were added, and vacuum–N_2 was applied. Then, toluene (5 mL) was added and degassed for 1 hour. After that, azetidine (0.08 g, 1.34 mmol, 3.5 eq.) was added and heated to 110 °C and stirred for 16 hours. After the completion of the reaction, the reaction mixture was cooled to room temperature and filtered through celite, then washed with $CHCl_3$. The crude product was purified with column chromatography (alumina, hexane:$CHCl_3$ 2:1),

and the target product, compound **6**, was obtained as an off-white solid (0.12 g) and used in next step without further purification.

Synthesis of **NSeMorph**

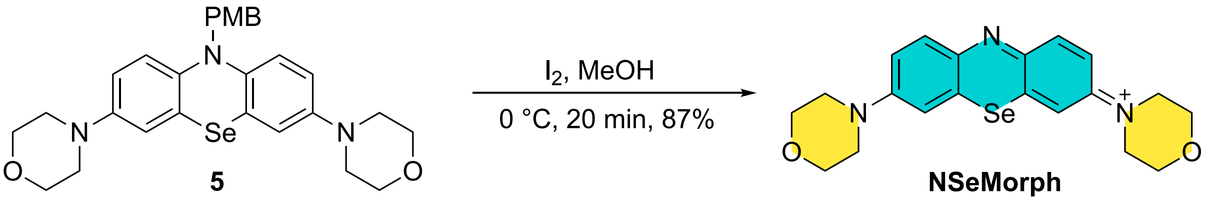

To a 50 mL two-necked round-bottom flask, compound **5** (55 mg, 0.10 mmol) was added, and vacuum–N_2 was applied. Then, it was dissolved in MeOH (10 mL). After that, reaction mixture was cooled to 0 °C and subsequently I_2 (50 mg, 0.20 mmol, 2 eq.) and stirred at that temperature for 20 min. After completion of reaction, mixture filtered and washed with hexane (200 mL) and greenish black solid was obtained (36 mg, **87%**). The target product, **NSeMorph**, purified with preparative HPLC. ^{1}H NMR (400 MHz, DMSO) δ 8.07 (d, J = 2.2 Hz, 2H), 8.00 (d, J = 9.6 Hz, 2H), 7.63 (dd, J = 9.7, 2.3 Hz, 2H), 3.87 – 3.83 (m, J = 4.6 Hz, 8H), 3.81 – 3.78 (m, J = 4.4 Hz, 8H). ^{13}C NMR (100 MHz, DMSO) δ 152.7, 139.9, 136.4, 135.4, 118.8, 110.8, 65.9, 47.8. HRMS calculated for $C_{20}H_{22}N_3O_2Se$: 416.0877. Found: 416.0877

Synthesis of **NSeAze**

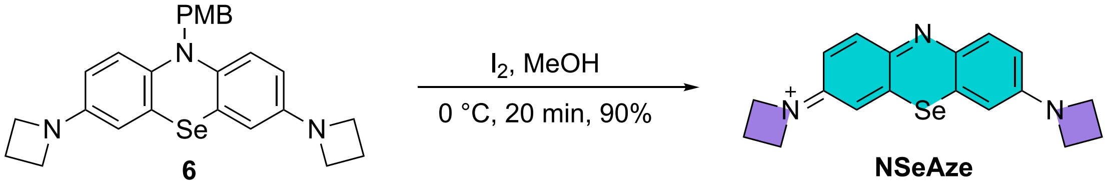

To a 50 mL two-necked round-bottom flask, compound **6** (50 mg, 0.10 mmol) was added, and vacuum–N_2 was applied. Then, it was dissolved in MeOH (10 mL). After that, reaction mixture was cooled to 0 °C and subsequently I_2 (51 mg, 0.20 mmol, 2 eq.) and stirred at that temperature for 30 min. After completion of reaction, mixture filtered and washed with hexane (200 mL) and greenish black solid was obtained (32 mg, **90%**). The target product, **NSeAze**, purified with preparative HPLC. ^{1}H NMR (400 MHz, DMSO) δ 7.86 (d, J = 9.3 Hz, 2H), 7.38 (d, J = 2.3 Hz, 2H), 6.96 (dd, J = 9.3, 2.3 Hz, 2H), 4.33 (t, J = 7.3 Hz, 8H), 2.49 – 2.43 (m, 4H). ^{13}C NMR (100 MHz, DMSO) δ 152.2, 139.7, 134.7, 134.4, 116.5, 108.5, 52.4, 15.4. HRMS calculated for $C_{18}H_{18}N_3Se$: 356.0666. Found: 356.0667

HPLC Analysis

Reverse phase HPLC analyses were conducted using Agilent Technologies 1260 Infinity II series HPLC systems with DAD detector. All the analyses were performed by using gradient elution with different Milli Q (0.1% TFA) and acetonitrile (0.8% TFA) systems as mobile phase and the column compartment temperature was 40°C. HPLC purifications were performed using a Phenomenex Kinetex 5 μm C18 100 A semi-preparative column with a flow rate of 1.5 mL/min. And for the purities, an Agilent Technologies Poroshell 120 EC-C18 analytical column was used with a flow rate of 0.5 ml/min.

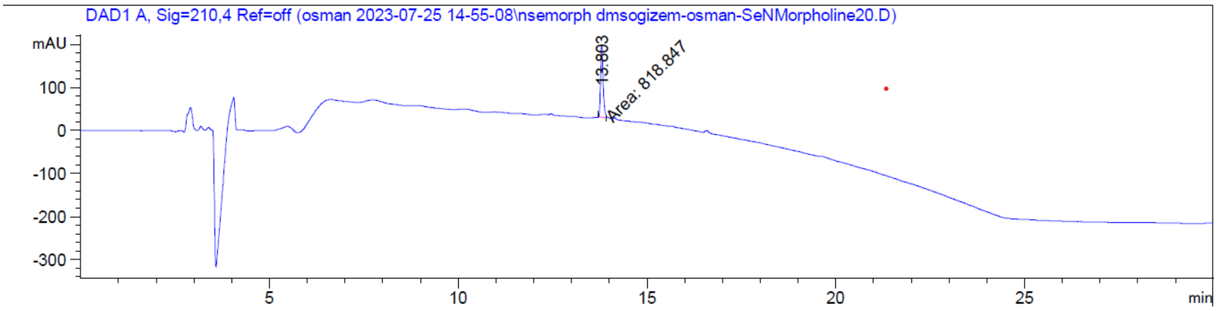

Figure S1. HPLC chromatogram of **NSeMorph**.

Table S1. HPLC retention times and peak areas of **NSeMorph**.

Peak	Ret. Time (min)	Area (%)
1	13.803	100.0

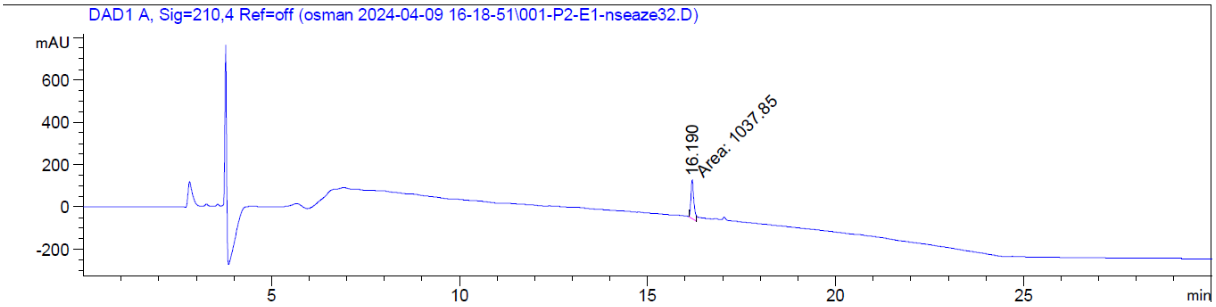

Figure S2. HPLC chromatogram of **NSeAze.**

Table S2. HPLC retention times and peak areas of **NSeAze**.

Peak	Ret. Time (min)	Area (%)
1	16.190	100.0

Photophysical Characterization

Fluorescence Quantum Yield

Fluorescence quantum yields of the samples were investigated using a fluorescence spectrometer (FLS 1000, Edinburgh Instruments) equipped with an integrating sphere accessory. A continuous-wave xenon lamp served as the excitation source, and the emitted fluorescence was detected with a standard photomultiplier (PMT-900) covering a wavelength range of 200-800 nm. During measurements, the PMT was cooled to -20 °C using a built-in housing to reduce undesired dark current noise.

For quantum yield measurement, an integrating sphere (Edinburgh Instruments) was placed inside the spectrometer's sample compartment. The internal cavity of the sphere was coated with a PTFE-like material, enabling a reflectance of approximately >99% (>95%) over the wavelength ranges of 400-1500 nm and 250-2500 nm, respectively. The sphere had two ports positioned 90° apart. The excitation beam was directed to the sample through the excitation port, and the fluorescence was collected from the emission port. The excitation port of the sphere included a lens to effectively focus the beam on the sample, while the emission port was an open aperture.

Prior to the experiments with the PSs, blank spectra were measured using the reference solvents (PBS, pH 7.4, 1% DMSO). For both measurements (blank and sample), two identical quartz cuvettes with equal volumes were used. Initially, the reference sample was placed inside the sphere, and the emission/excitation slits were adjusted to the excitation wavelength to ensure the PMT's response remained linear during measurements. To cover a scattering range, the emission scans started 20 nm below the actual excitation wavelengths and ended at 900 nm. Additionally, the step size and integration time of the measurements were set to 1 nm and 0.2 seconds, respectively.

Upon completing all emission measurements of the samples and references, the quantum yields of the samples were determined using the Fluoracle software. The built-in analysis tool calculates the quantum yield (QY) as:

$$QY=(Es-EB)/(SB-Ss) ,$$

where Es (EB) and Ss (SB) are the selected areas for the emitted and scattered signals of the sample (blank).

Singlet Oxygen Trap Experiment

Singlet oxygen quantum yields were calculated using the following equation and methylene blue as the reference fluorophore. The singlet oxygen trapping experiment for methylene blue was performed at pH 7.4 in phosphate saline buffer, with a reported yield of 0.52 from the literature. The relative quantum yields were determined with reference to methylene blue in phosphate saline buffer. Oxygen-saturated PBS was prepared by bubbling molecular oxygen through the solution for 10 minutes. The cuvette was then filled with a 5 µM photosensitizer solution, corresponding to an absorbance of approximately 0.2-0.3. 9,10-Anthracenediyl-bis(methylene)dimalonic acid (ADMDA) was added as a singlet oxygen trap, and its absorbance was adjusted to between 0.6 and 1.9.

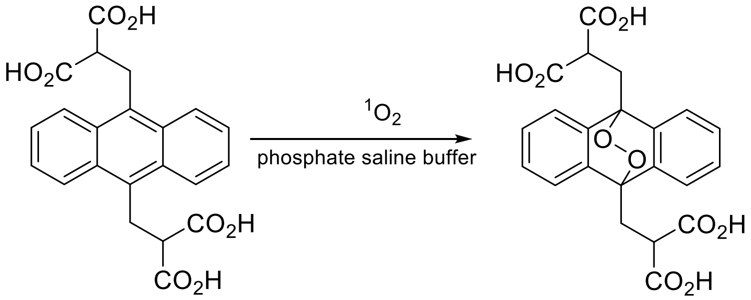

Figure S3. Reaction between photosensitized $^1O_2$ and ADMDA.

Several measurements were taken in the dark to stabilize the trap's solubility. Subsequently, the cuvette was exposed to light of an appropriate wavelength. Each irradiation was performed from a distance of 10 cm for 30 seconds durations. The decrease in the trap's absorbance at 380 nm was recorded and repeated multiple times. The slope for each photosensitizer was calculated by plotting the trap’s absorbance at 380 nm versus time. Singlet oxygen quantum yields were then calculated according to the following equation:

$$\Phi_{\Delta sample} = \Phi_{\Delta standard} \left(\frac{1 - 10^{-A_{std}}}{1 - 10^{-A_{sam}}}\right)\left(\frac{m_{sample}}{m_{standard}}\right)$$

Here, "sample" and "standard" represent photosensitizer derivatives and methylene blue, respectively. m is the slope for the photosensitizer derivatives, indicating the variation of the trap's absorbance at 380 nm with irradiation time, A_{sam} and A_{std} represents F·PF for PS and methylene blue respectively, F is the correction factor,

which is given by F = 1 – 10^{-OD} (OD at absorption maxima of irradiation wavelength), and PF is absorbed photonic flux in μEinstein $dm^{-3}s^{-1}$.

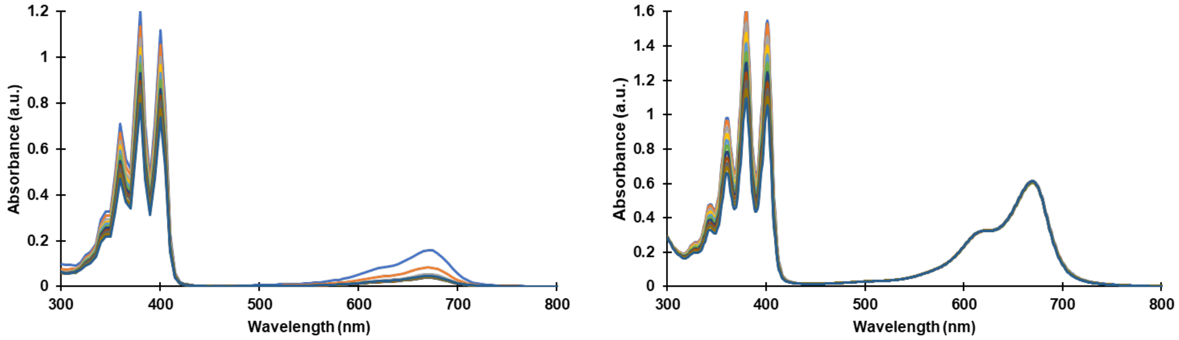

Figure S4. Decrease in the absorbance of ADMDA in PBS (pH 7.4, 1% DMSO) upon irradiation of **NSeMorph** (20 μM) (left) and **NSeAze** (20 μM) (right).

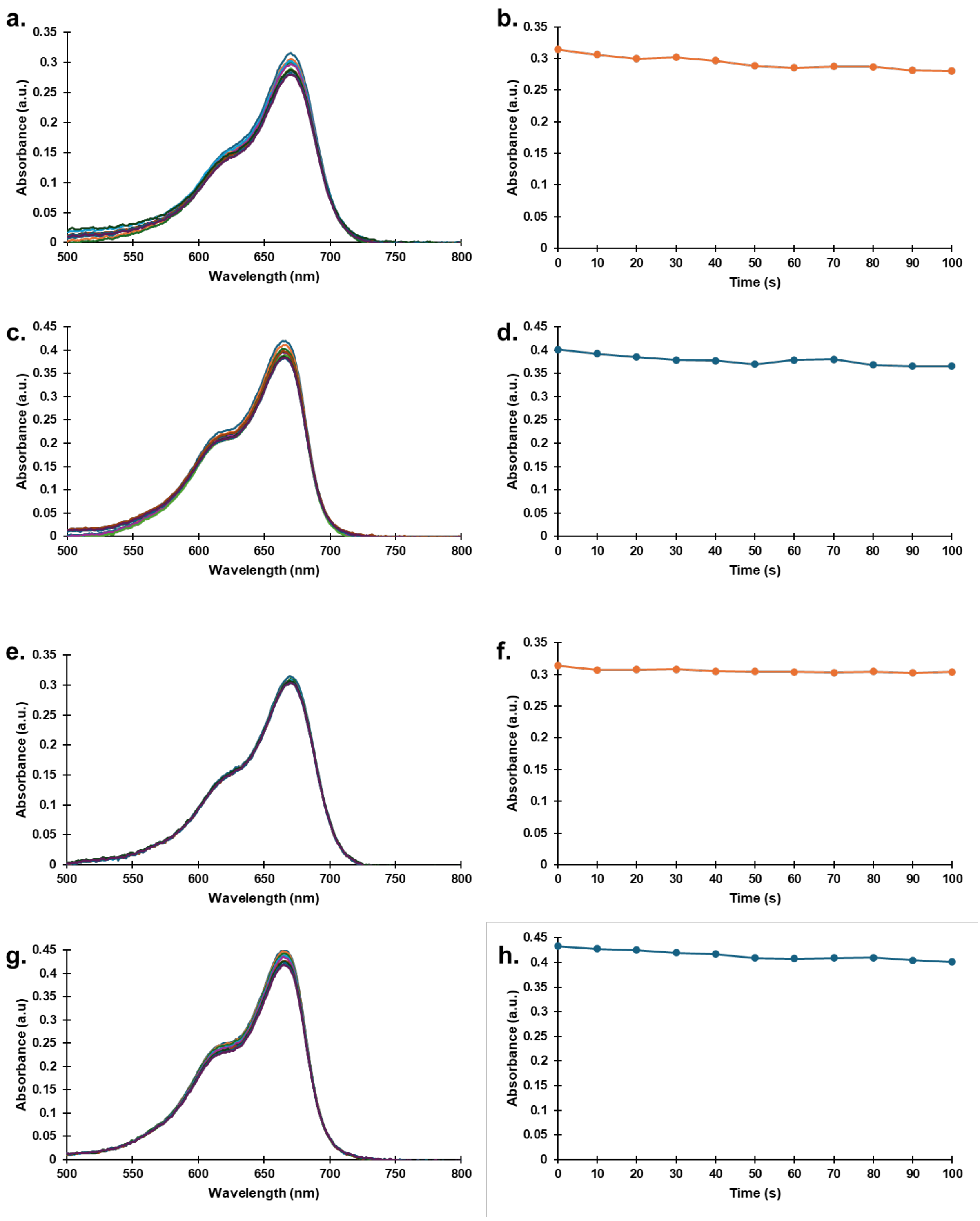

Figure S5. Photostability of **NSeMorph** (20 μM) in sodium citrate buffer (1% DMSO) **a.** pH 5.4 and **e.** pH 6.4 upon irradiation with LED light (660 nm, 100 s total irradiation time) and its time dependent absorbance profile at **b.** pH 5.4, **f.** pH 6.4. Photostability of **NSeAze** (20 μM) in sodium citrate buffer (1% DMSO) **c.** pH 5.4 and **g.** pH 6.4 upon irradiation with LED light (660 nm, 100 s total irradiation time) and its time dependent absorbance profile at **d.** pH 5.4, **h.** pH 6.4.

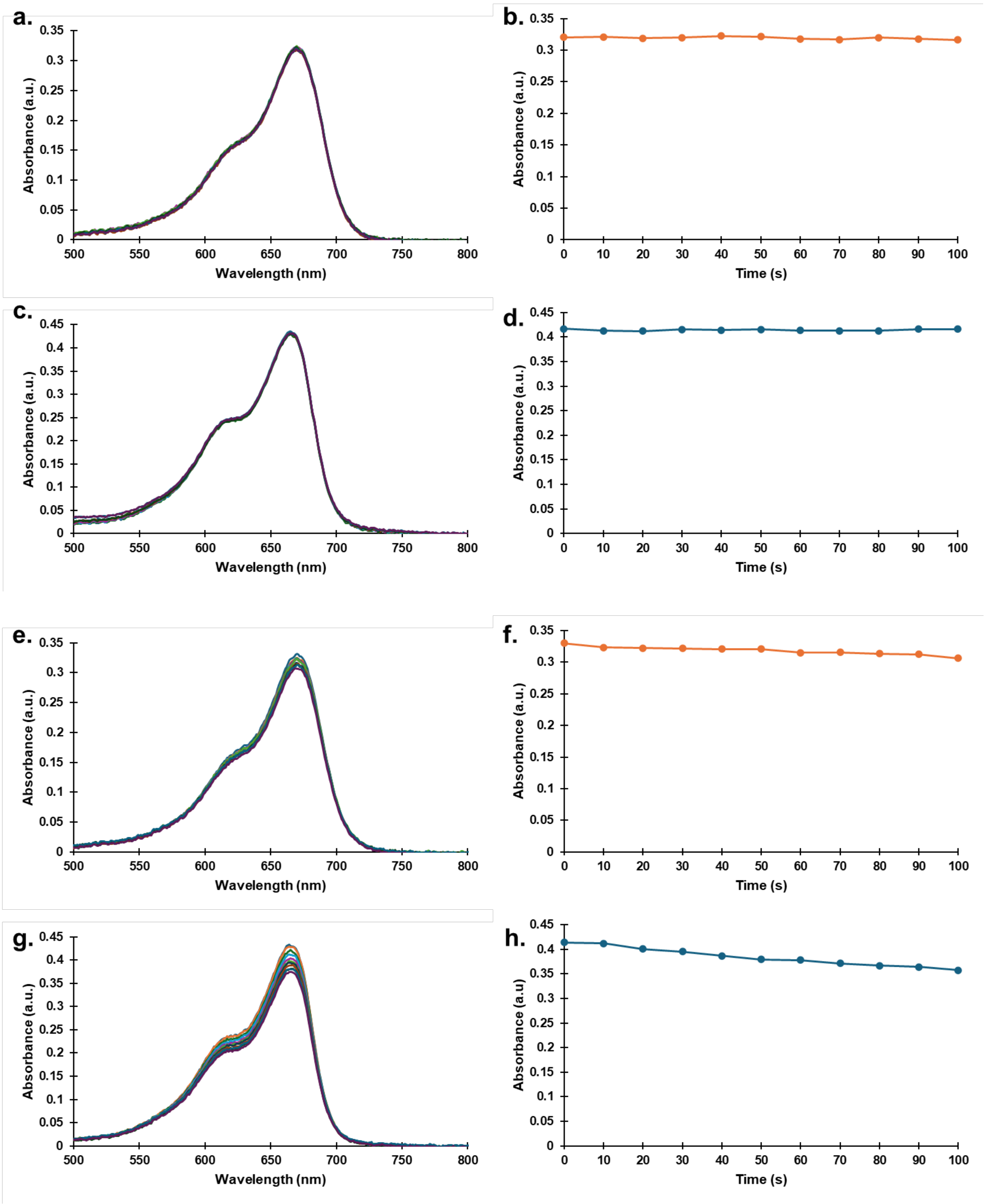

Figure S6. Photostability of **NSeMorph** (20 μM) in PBS buffer (1% DMSO) **a.** pH 7.4 and boric acid buffer **e.** pH 8.4 upon irradiation with LED light (660 nm, 100 s total irradiation time) and its time dependent absorbance profile at **b.** pH 7.4, **f.** pH 8.4. Photostability of **NSeAze** (20 μM) in PBS buffer (1% DMSO) **c.** pH 7.4 and boric acid buffer **g.** pH 8.4 upon irradiation with LED light (660 nm, 100 s total irradiation time) and its time dependent absorbance profile at **d.** pH 7.4, **h.** pH 8.4.

SOSG Oxidation in the Presence of $^1O_2$

The reaction between Singlet Oxygen Sensor Green (SOSG) and singlet oxygen ($^1O_2$), generated by photoirradiation of **NSeMorph** or **NSeAze** in PBS buffer containing 1% DMSO, was investigated. The concentration of SOSG was maintained at 1 μM, and the photosensitizer (PS) concentration was kept at 40 μM. Fluorescence measurements were carried out using a spectrofluorometer with excitation and emission wavelengths set at 488 nm and 525 nm, respectively. The cuvette containing the PS and SOSG was exposed to 660 nm LED light. Each irradiation was performed from a distance of 10 cm for durations of 10 seconds. The increase in SOSG fluorescence intensity at 525 nm was monitored for up to 100 seconds by the fluorescence spectrofluorometer.

DHR Oxidation for Detection of Superoxide anion $O_2^{\bullet -}$

The generation of superoxide anion ($O_2^{\bullet -}$) was detected using DHR123 in a phosphate-buffered saline (PBS, pH 7.4) solution. Upon formation of $O_2^{\bullet -}$, DHR123 undergoes oxidation, resulting in a strong emission signal centered at 526 nm. Solutions were prepared by mixing 3 mL of PBS containing either **NSeMorph** or **NSeAze** (40 μM) with DHR123 (1 μM). The mixture was then transferred to a cuvette and irradiated with 660 nm LED light. Irradiation was performed from a distance of 10 cm for 10-second intervals. The increase in DHR123 fluorescence intensity at 526 nm was monitored for up to 100 seconds using a fluorescence spectrofluorometer.

Cell Culture and Treatment

Human glioblastoma cell lines (U118MG and U87MG) and healthy mouse fibroblast cells (L929) were cultured in DMEM high glucose medium supplemented with 10% fetal bovine serum (FBS), 2 mM glutamine, 1% penicillin/stremptomycin, 0.5% amphotericin B, and incubated at 37 °C with 5% CO_2. **NSeMorph** and **NSeAze** were dissolved in cell culture-grade DMSO and further diluted in complete medium for experimental use. For phototoxicity analysis, cells were administered with **NSeMorph** (0.5-20 μM) and **NSeAze** (0.01-2.5 μM) for 0.5−4 h followed by 660 nm LED (24.3 mW/cm^2) irradiation for 2 h in **NSeMorph**-treated groups and 1 h in **NSeAze**-treated groups. Then, each group were incubated for further 24 h in the dark to allow for resting. On the other hand, the dark toxicity of both agents was detected following the same treatment under identical conditions without LED irradiation. To determine the involvement of distinct ROS types, cells were pretreated for 1 h with selective

scavengers prior to photosensitizer exposure. The scavengers included NAC (5 mM), NaN_3 (5 mM), histidine (5 mM), Tiron (100 µM), mannitol (25 mM) and Trolox (25 µM). Cell viability was then assessed following irradiation. For index calculations, all cells were treated with the increasing concentrations (0.5-500 µM) of **NSeMorph** and **NSeAze** for 24 h. Final DMSO concentrations were kept below 0.5% (v/v) in all treatments.

PI=$IC_{50,\ dark}$ /$IC_{50,\ light}$
SI= $IC_{50,\ light,\ healthy\ cells}$/$IC_{50,\ light,\ cancer\ cells}$
TI= $IC_{50,\ dark,\ healthy\ cells}$/$IC_{50,\ light,\ cancer\ cells}$

Cell Viability Analysis

The viability of cells treated with **NSeMorph** and **NSeAze** was determined with MTT (3-[4,5-dimethylthiazol-2-yl]-2,5-diphenyltetrazoliumbromide) assay. Following the administration of both compounds, the medium was discarded and cells were treated with fresh media containing MTT (0.5 mg/ml) for 2-4 h at 37 °C. Formazan crystals were then solubilized in 10% SDS in 0.01 N HCl, and absorbance was measured at 490 and 570 nm wavelength using microplate reader (Multiskan Sky, Thermo Scientific). Data were normalized to vehicle (DMSO)-treated controls. IC_{50} values were calculated using nonlinear regression analysis in GraphPad Prism 9.02 (GraphPad Software Inc.). All experiments were performed in technical and biological replicates **(n = 6).**

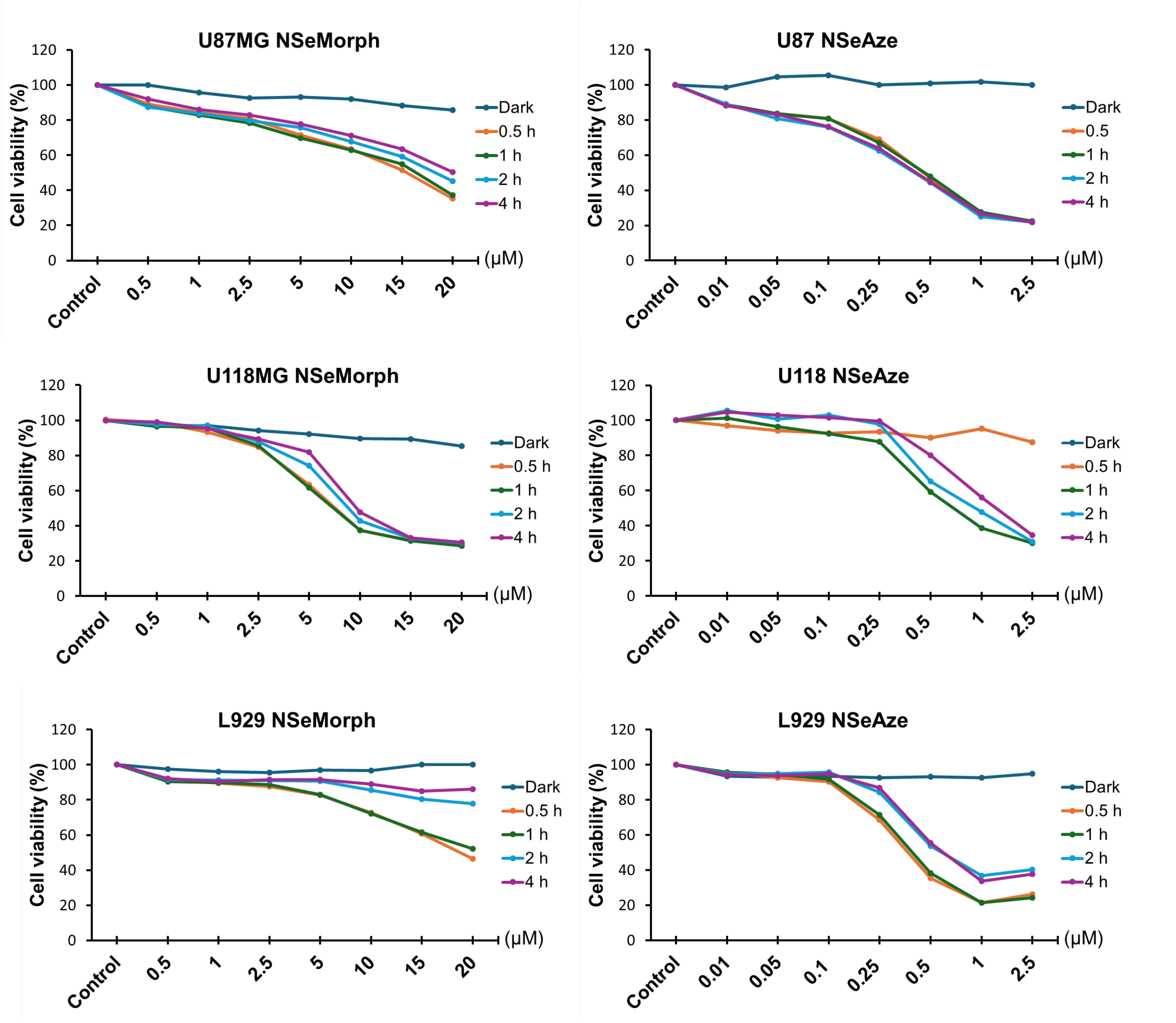

Figure S7. The time- and concentration-dependent effect of **NSeMorph** and **NSeAze** on U87MG, U118MG glioblastoma and L929 healthy cells. Both compounds were administered in a range of concentrations as indicated. For dark toxicity 24 h of incubation was carried out, whereas for PDT application cells were incubated for 0.5, 1, 2 and 4 h prior to irradiation. **NSeMorph**-treated cells were exposed to 2 h of LED irradiation and **NSeAze**-treated cells were exposed to 1 h of irradiation, followed by 24 h dark incubation. The resulting viability of each group was compared to vehicle control and expressed as %.

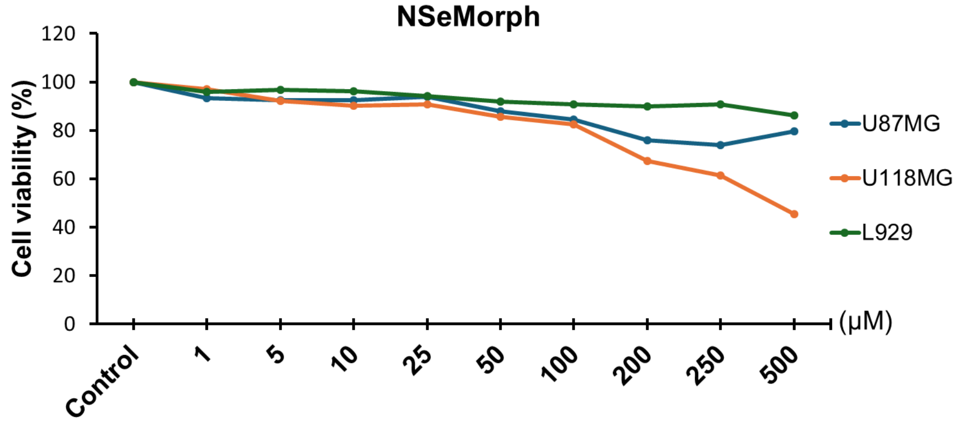

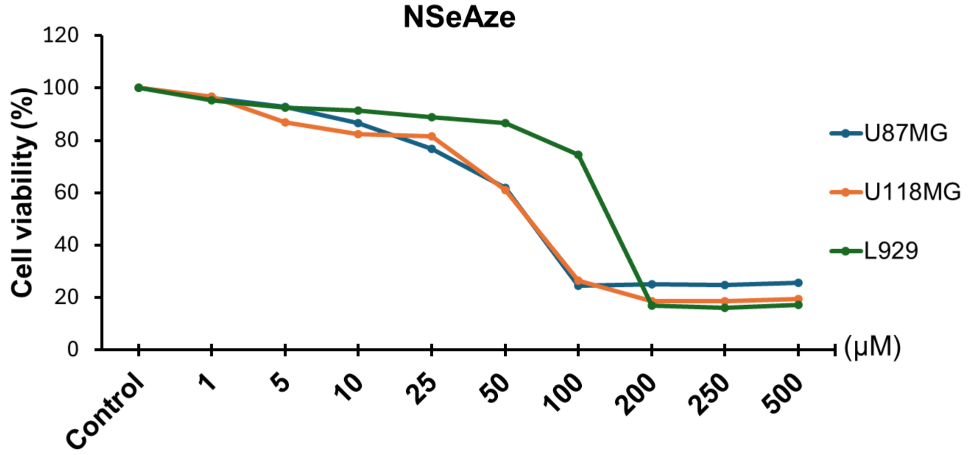

Figure S8. The effect of **NSeMorph** and **NSeAze** on U87MG, U118MG glioblastoma and L929 healthy cells in higher concentrations. Both compounds were administered in the dark for 24 h. The resulting viability of each group was compared to vehicle control and expressed as %.

Cellular Uptake Assay

To quantify the intracellular accumulation of the photosensitizers, U87MG, U118MG, and L929 cells were seeded into 96-well plates at a density of 5 × 10^4 cells/well and incubated overnight in DMEM supplemented with 10% FBS under standard culture conditions 37 °C, 5% CO_2. The next day (90-100% confluency), cells were treated with 100 μM of **NSeAze** or **NSeMorph** in 100 μL of complete medium and incubated for 1 hour. After treatment, the medium was removed, and cells were washed once with 100 μL PBS to remove excess amount of PS. Subsequently, 50 μL of DMSO was added to each well to lyse the cells and solubilize the photosensitizers. Plates were placed on a shaker for 10-15 minutes at room temperature to ensure complete release. The absorbance values were measured at 671 nm wavelength and normalized to vehicle treated controls (n=3).

Cell Uptake (%) = [(A_{well} − A_{blank}) × V_{DMSO})]/ [ε × l × C_0 × V_0)] × 100

A_{well} = absorbance of the sample well

A_{blank} = absorbance of the blank well

V_{DMSO} = volume of DMSO used for extraction (in liters)

ε = molar extinction coefficient of the PS at 671 nm (in $M^{-1}\cdot cm^{-1}$)

l = path length (0.14 cm)

C_0 = initial concentration of PS added to the cells (in M)

V_0 = volume of applied PS solution (in liters)

Confocal Imaging Studies

Cellular internalization

For cellular internalization studies, U87MG and U118MG cells were seeded in black 24-well plates (3× 10^4 cells/well) and incubated overnight. Cells were treated with **NSeAze** or **NSeMorph** at 2.5 μM concentration for 0.5, 1, 2, and 4 h, then washed twice with PBS. Cells were fixed with 4% paraformaldehyde for 15-20 min at RT. After washing steps, cells were stained with Hoechst 33342 (0.5 μg/ml). Images were captured using a 40X objective, and exposure parameters were kept constant across time points. The fluorescence intensities of the photosensitizers were normalized relative to the 0.5 h time point to evaluate time-dependent internalization. Each experimental condition was performed in triplicate (n = 3).

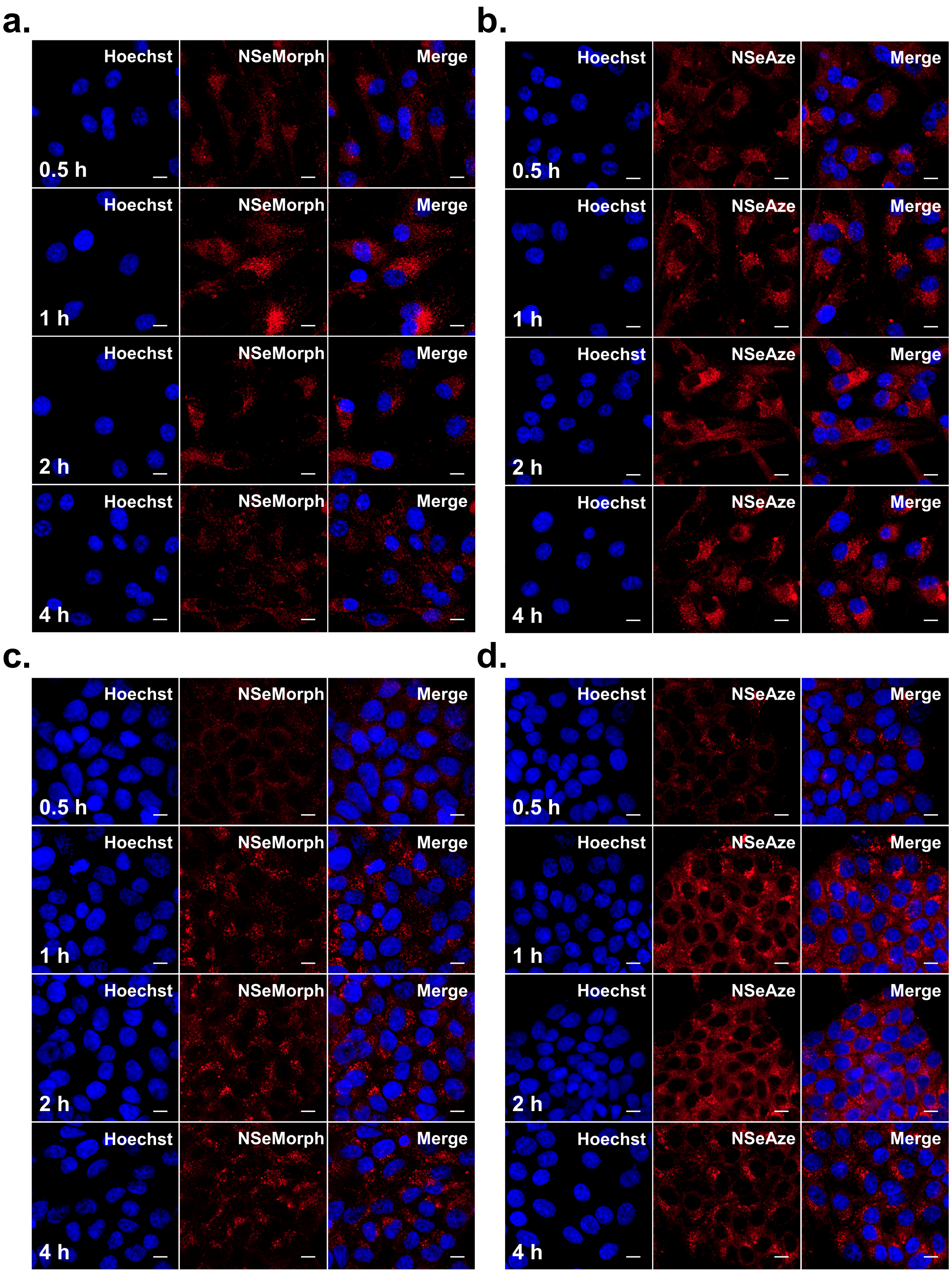

Figure S9. Time-dependent internalization of **NSeMorph** (2.5 μM) and **NSeAze** (2.5 μM) incubated for 0.5, 1, 2 and 4 h in U87MG **(a,b)** U118MG **(c,d)** cells. Blue, Hoechst 33342; Red, **NSeMorph** or **NSeAze.** Scale bar: 10 μm

Subcellular co-localization

Cells were seeded in 35 mm glass-bottom confocal dishes (2 × 10^4 cells/well) and treated with **NSeAze** or **NSeMorph** (2.5 μM) for 0.5, 1, 2, or 4 h. After incubation periods, cells were washed with PBS and incubated with the following organelle-specific trackers: MitoTracker™ Green FM (50 nM, 30 min), LysoTracker™ Yellow HCK-123 (75 nM, 45 min), and Hoechst 33342 (0.5 μg/mL, 15 min). Imaging was performed using a Zeiss LSM 900 confocal laser scanning microscope (CLSM). Excitation/emission wavelengths were: Hoechst, 361/497 nm; MitoTracker, 488/516 nm; LysoTracker, 465/535 nm; PS fluorescence was collected in the red channel (Cy5.5; 570–620 nm).

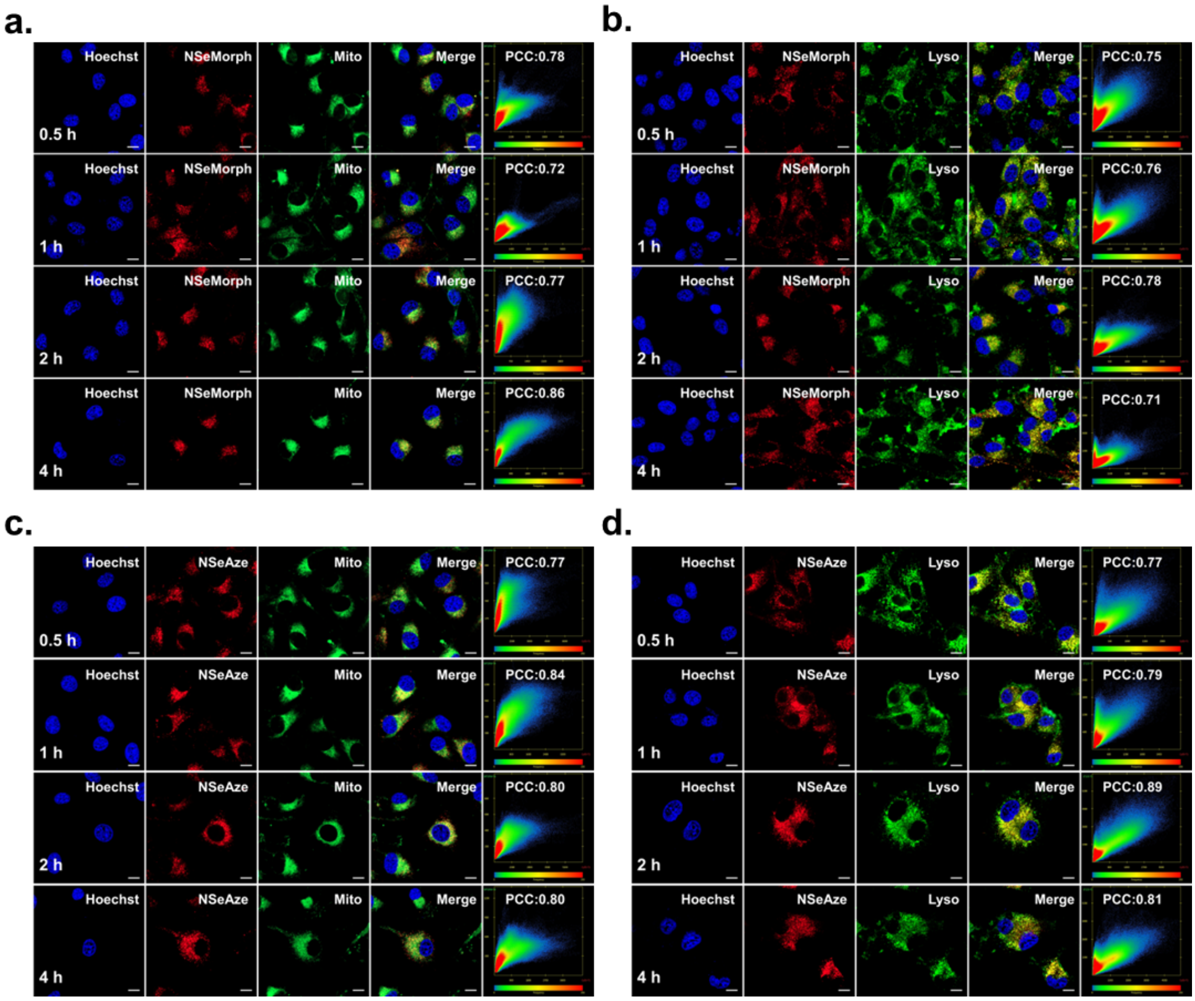

Figure S10. Time-dependent subcellular co-localization of **NSeMorph** (2.5 μM) **(a,b)** and **NSeAze** (2.5 μM) **(c,d)** incubated for 0.5, 1, 2 and 4 h in **U87MG** cells. Blue, Hoechst 33342; Red, **NSeMorph** or **NSeAze**; Green, Mitotracker or Lysotracker. Scale bar: 10 μm

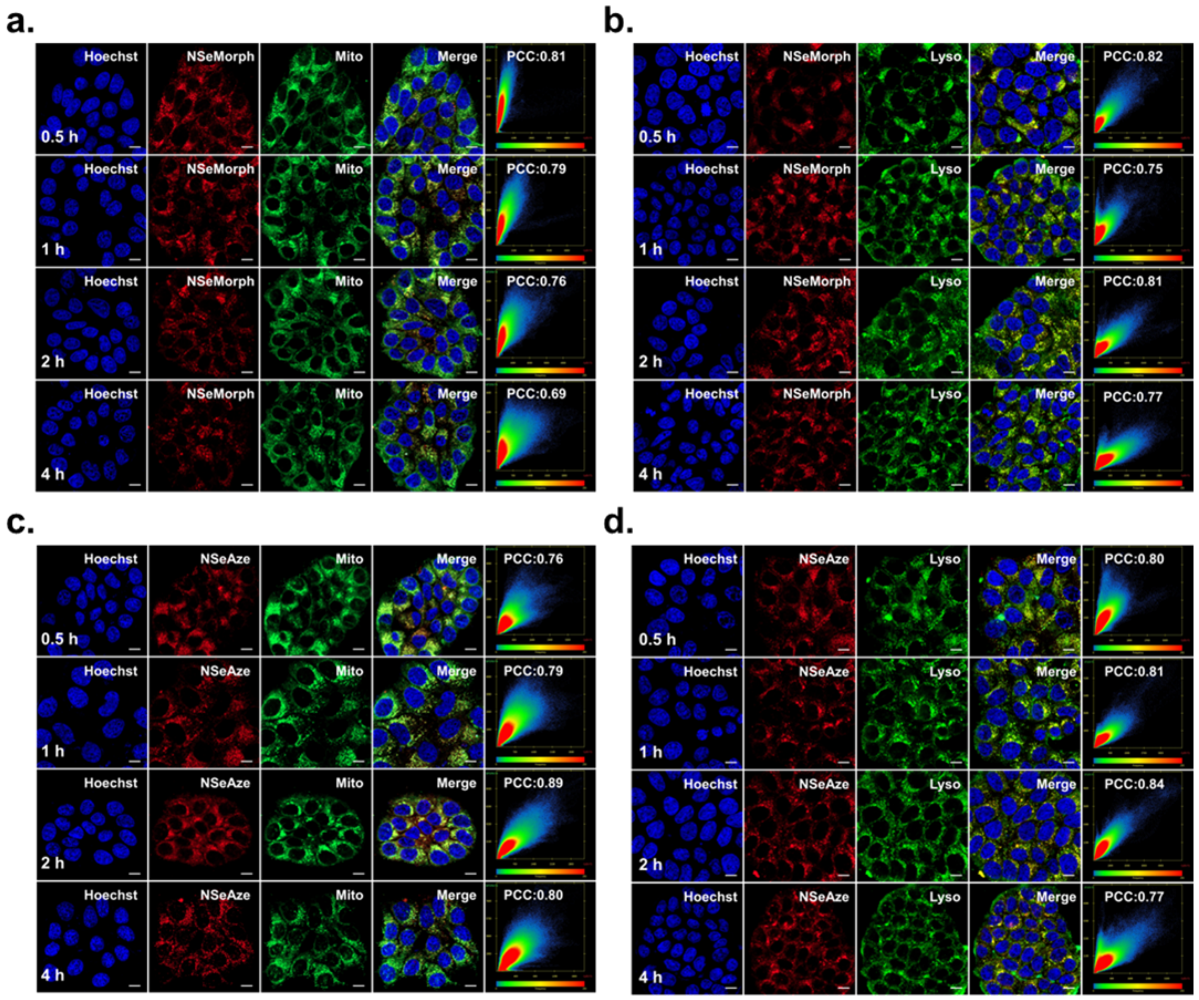

Figure S11. Time-dependent subcellular co-localization of **NSeMorph** (2.5 μM) (a,b) and **NSeAze** (2.5 μM) (c,d) incubated for 0.5, 1, 2 and 4 h in **U118MG** cells. Blue, Hoechst 33342; Red, **NSeMorph** or **NSeAze**; Green, Mitotracker or Lysotracker. Scale bar: 10 μm

To ensure comparability across time points, the fluorescence intensities of the photosensitizers were kept consistent by adjusting image processing parameters uniformly. Co-localization was evaluated by calculating Pearson's correlation coefficients (PCCs) using the co-localization analysis tool in Zeiss Zen Blue software, comparing red channel intensity with organelle-specific green signals. All experimental conditions were conducted in triplicate (40X, n = 3).

Reactive oxygen species (ROS) detection

ROS generation assay was performed using DCFH-DA (2′,7′-dichlorofluorescin diacetate) probe via confocal microscopy. In brief, U87MG and U118MG glioblastoma cells were seeded in 96-well plates at a density of 3 × 10^4 cells/well and allowed to adhere overnight under standard culture conditions. Cells were then treated with IC_{50}

values of **NSeMorph** or **NSeAze** for 1 h in the dark, followed by exposure to 660 nm LED (24.3 mW/cm^2) irradiation for photodynamic activation.

To assess the role of specific ROS types, parallel groups were co-incubated with selective scavengers added 1 hour prior to the PS treatment. The following scavengers were used at established effective working concentrations: N-acetylcysteine (NAC, 5 mM) as a broad-spectrum ROS and thiol-based antioxidant; sodium azide (NaN_3, 5 mM) and histidine (5 mM) for quenching singlet oxygen ($^1O_2$); Tiron (100 μM) for superoxide anion ($O_2^{\bullet-}$); mannitol (25 mM) for hydroxyl radicals ($HO^\bullet$); and Trolox (25 μM) for peroxyl radicals ($ROO^\bullet$). The following scavengers were used at established effective working concentrations: N-acetylcysteine (NAC, 5 mM) as a broad-spectrum ROS and thiol-based antioxidant; sodium azide (NaN_3, 5 mM) and histidine (5 mM) for quenching singlet oxygen ($^1O_2$); Tiron (100 μM) for superoxide anion ($O_2\bullet^-$); mannitol (25 mM) for hydroxyl radicals ($HO^\bullet$); and Trolox (25 μM) for peroxyl radicals ($ROO^\bullet$). After treatment, cells were washed twice with 1XPBS and incubated with DCFH-DA (20 μM) and Hoechst 33342 (0.5 μg/mL) in serum-free medium for 20-30 minutes at 37 °C. Stained cells were washed again with PBS, then fresh-serum free medium was added. Imaging was performed using a Zeiss LSM 900 confocal laser scanning microscope at 488/535 nm (ex/em) for DCF fluorescence and 361/497 nm (ex/em) for Hoechst nuclear stain, using a 10X objective. Representative fields from each condition were imaged and compared for intracellular ROS signal intensity and scavenger-mediated inhibition. All conditions were analysed in biological triplicates (n = 6).

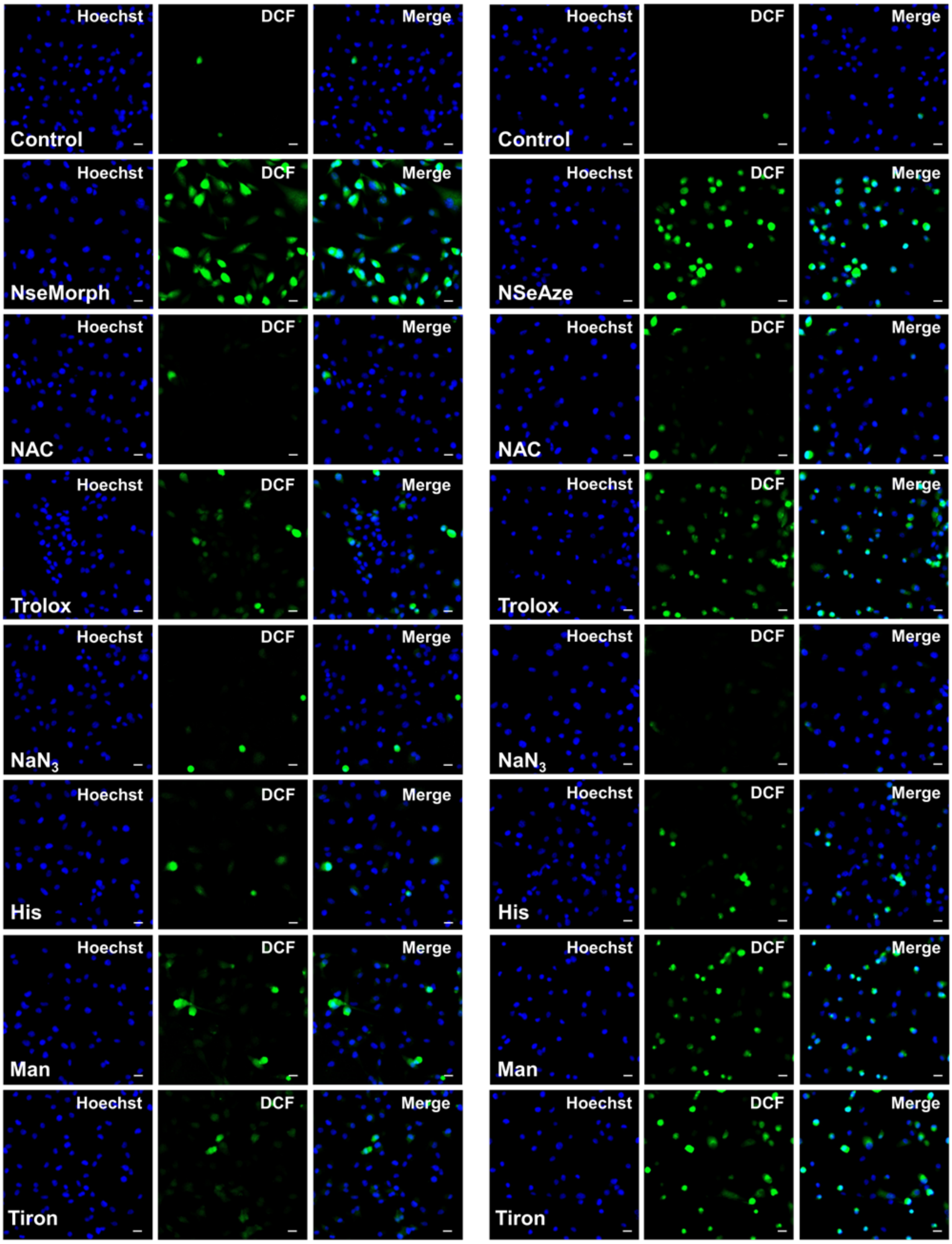

Figure S12. The effect of **NSeMorph** and **NSeAze** on ROS generation in **U87MG** cells. Cells were treated with IC_{50} values of both compounds in the presence or absence of scavengers and confocal images were acquired following Hoechst and DCFH-DA staining. Scale bar: 20 µm. Blue, Hoechst 33342; green, DCF. NAC: N-acetylcysteine, His: Histidine, Man: Mannitol

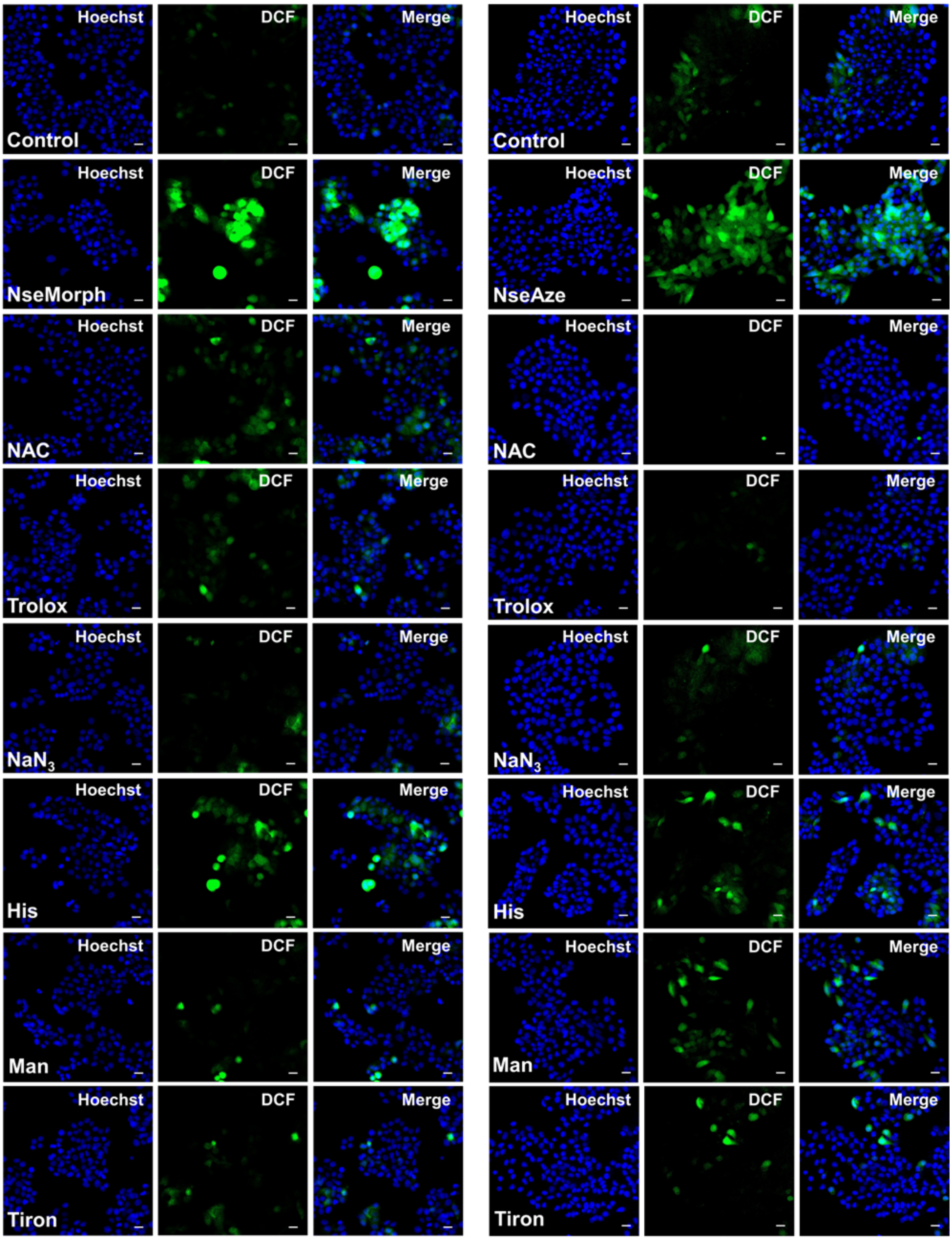

Figure S13. The effect of **NSeMorph** and **NSeAze** on ROS generation in **U118MG** cells. Cells were treated with IC_{50} values of both compounds in the presence or absence of scavengers and confocal images were acquired following Hoechst and DCFH-DA staining. Scale bar: 20 µm. Blue, Hoechst 33342; green, DCF. NAC: N-acetylcysteine, His: Histidine, Man: Mannitol

Mitochondrial superoxide detection

Mitochondrial superoxide generation was assessed using the dihydrorhodamine 123 (DHR123) fluorescent probe. After incubation with the IC_{50} concentrations of NSeMorph or NSeAze for 1 hour in the dark, DHR123 (10 μM) was added to the medium during the 660 nm LED (24.3 mW/cm^2) irradiation period and maintained throughout the light exposure to enable real-time detection of mitochondrial ROS production. Cells were irradiated in the presence or absence of ROS scavengers, which had been pre-applied 1 hour prior to PS treatment and retained throughout the irradiation phase. Following irradiation, cells were washed twice with 1X PBS, counterstained with Hoechst 33342 (0.5 μg/mL) for 15 minutes, and washed again with PBS. Imaging was conducted at 508/536 nm ex/em for DHR123 and 361/497 nm for Hoechst, with a 10X objective. Representative fields were captured from each group to compare mitochondrial ROS fluorescence intensities between untreated and scavenger-treated conditions. All experimental groups were performed in biological triplicates (n = 6). Similar experiment with single treatments was performed under dark and light conditions and visualized with CLSM at 40X objective (n=3).

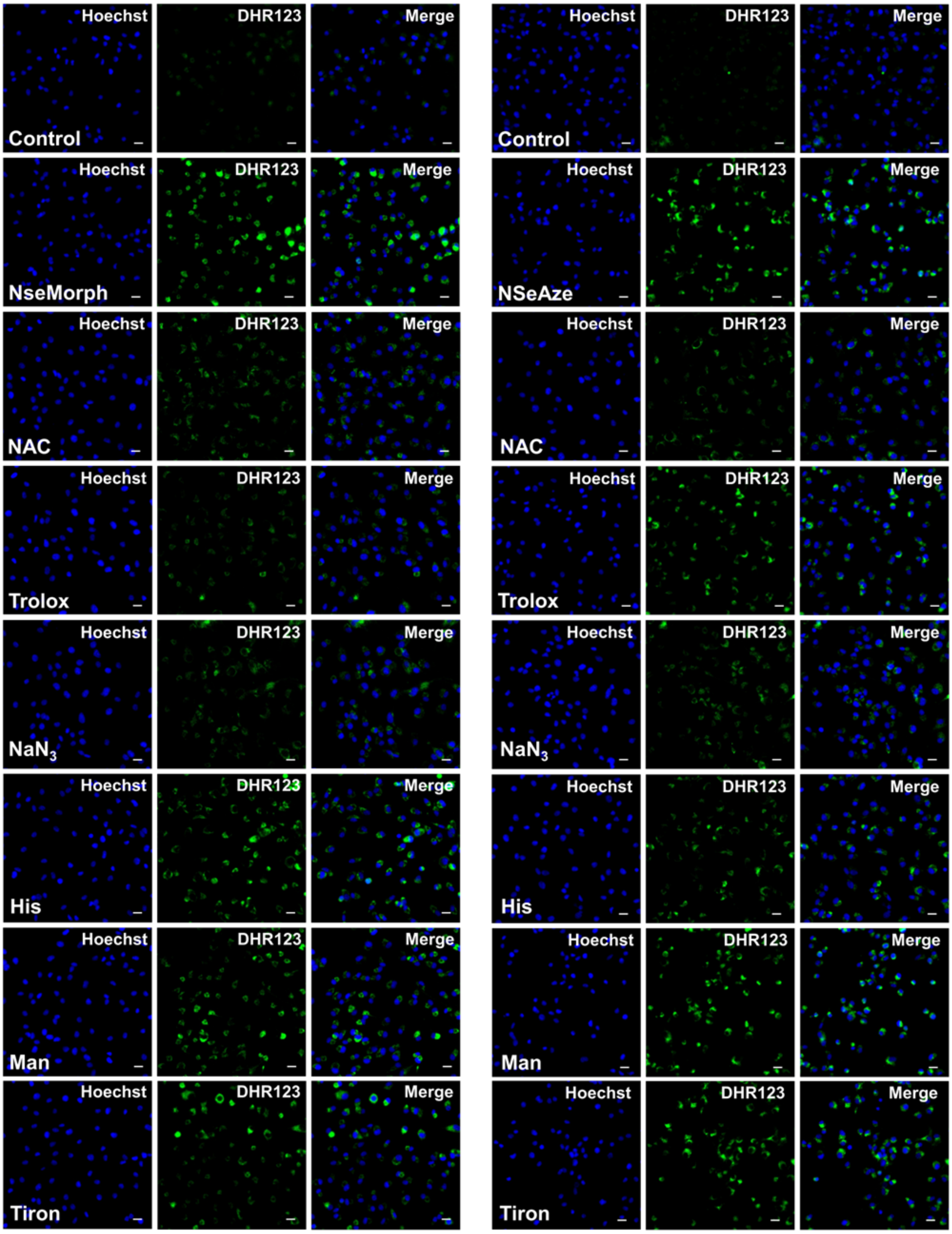

Figure S14. Mitochondrial ROS generation in **U87MG** cells with dihydrorhodamine 123 (DHR123) staining. Cells were treated with IC_{50} values of **NSeMorph** and **NSeAze** in the presence or absence of scavengers and confocal images were acquired following Hoechst and DHR123 staining. Scale bar: 20 μm. Blue, Hoechst 33342; green, DHR123. NAC: N-acetylcysteine, His: Histidine, Man: Mannitol

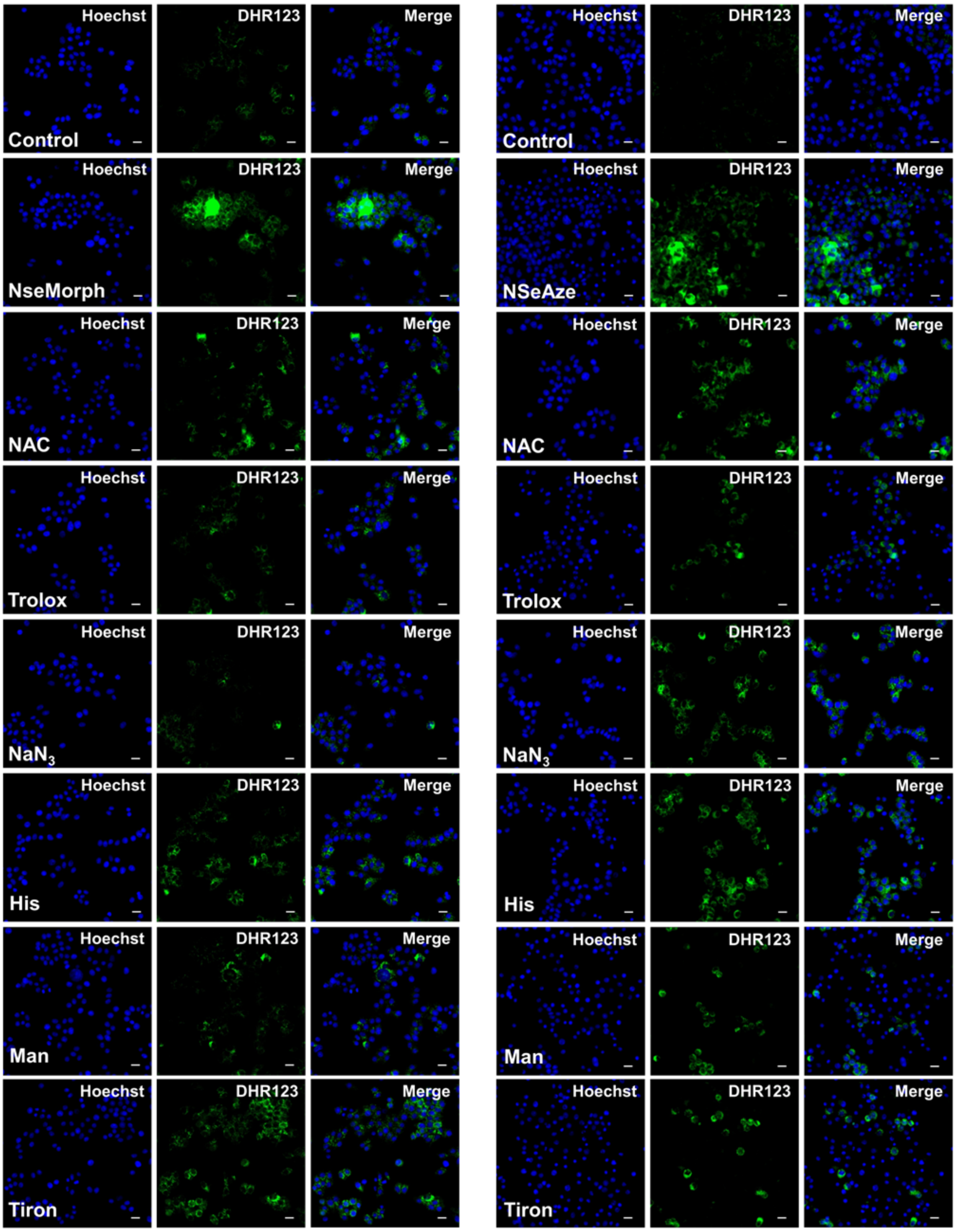

Figure S15. Mitochondrial ROS generation in **U118MG** cells with dihydrorhodamine 123 (DHR123) staining. Cells were treated with IC_{50} values of **NSeMorph** and **NSeAze** in the presence or absence of scavengers and confocal images were acquired following Hoechst and DHR123 staining. Scale bar: 20 µm. Blue, Hoechst 33342; green, DHR123. NAC: N-acetylcysteine, His: Histidine, Man: Mannitol

Determination of lipid peroxidation

The effect of **NSeMorph** and **NSeAze** on lipid peroxidation was investigated using thiobarbituric acid reactive substances (TBARS) method,[1] which is based on measuring malondialdehyde (MDA) levels, the end-product of lipid peroxidation. For this purpose, cells were seeded in 6-well plates at a density of 1 × 10^6 cells/well and incubated overnight. Afterwards, both compounds were administered in IC_{50} values according to the above-mentioned PDT protocol. The resulting lipid peroxidation was measured following the immediate irradiation and after 24 h of resting stage. After the indicated time periods, cells were harvested and lysed with a RIPA cell lysis buffer (Merck, Germany). The lysates were then prepared according to the TBARS assay protocol. Briefly, the samples were mixed with 0.8% thiobarbituric acid prepared in 20% trichloroacetic acid solution and then incubated for 2 h at 95ºC. Absorbance of the resulting pink-to-red color was measured at 532 nm with a spectrophotometer (Thermo, Germany). Data were expressed as % compared to untreated control group and normalized to cell viability rates (n=6).

Sulfo-phospho-vanillin assay

The intracellular lipid content following **NSeMorph** and **NSeAze** treatment was determined using sulfo-phospho-vanillin method which detects primarily unsatured free lipids.[2] Cell lysates were prepared as indicated above in lipid peroxidation experiment. The protein concentration of each sample was calculated using bicinchoninic acid (BCA) method according to the instructions of the manufacturer (Thermo Scientific, Germany). Briefly, 40 µl of cell lysates were mixed with 200 µl of concentrated H_2SO_4 and incubated for 10 mins at 90ºC. After cooling the samples, 120 µl of vanillin solution (1 mg/ml in 17% phosphoric acid) was added and the resulting red color was detected with a microplate reader at 520-540 nm wavelengths.[3] Data were expressed as % compared to untreated control group and normalized to protein concentration (n=6).

Acridine Orange/Ethidium Bromide (AO/EtBr) staining for cell death

Dual acridine orange/ethidium bromide (AO/EtBr) staining was performed to discriminate live, early apoptotic and/or necrotic cells on the basis of membrane permeability.[1,2] U87MG and U118MG cells were seeded in 24-well plates at a density of 5 × 10^4 cells/well and treated with the IC_{50} concentrations of the photosensitizers **NSeMorph** and **NSeAze**. After irradiation, cells were rested at 37 °C for 20 minutes, followed by staining with AO (2.5 µg/ml) and EtBr (2.5 µg/ml) in serum-free DMEM

under dark conditions for an additional 20-30 minutes. Subsequently, cells were washed with 1X PBS, and confocal images were taken at 500/525 nm (ex/em) for AO and 530/617 nm (ex/em) for EtBr (10X, n=6).

Table S3. Comparison of recently reported PSs for the treatment of glioblastoma multiforme.

Photosensitizer	Abs* (nm)	Mwt (g/mol)	IC_{50} (μM)	Cell Line	PI	Ref.
**Gp-Wm extract**	570	-	-	U87MG	-	[4]
**5-ALA-PpIX**	630	730.3	0.062<br>0.052	H-4 +Ko143<br>H-4 +Lap	-	[5]
**Se-718**	718 (DMSO)	418.4	0.32	U87MG	-	[6]
**^{mito}ZnPor**	~450	1954.7	0.045	U87MG	387	[7]
**BASHY-4**	511 (ACN)	1018.7	0.098	U87MG	>1000	[8]
**$Co(L)_2(H_2O)_2$**<br>**$Mn(L)_2(H_2O)_2$**	492<br>494<br>(DMF)	872.2<br>864.2	(μg/mL)<br>115<br>42	U87MG	-	[9]
**Benzoporphyrin derivative (BPD)**	692	718.8	13.12<br>11.56	U87MG<br>U251	-	[10]
**Curcumin (CUR)**	410	368.4	5.74	SNB-19	-	[11]
**RGD-$(Linker)_2$-Glu-SiPc**	674	1934.8	0.262	U87MG	-	[12]
**$[(Ph_2phen)_2Os(dpp)]_2^+$**	650	1379.2	86.07	F98	-	[13]
**PcAlCl in UDLs**	674 (EtOH)	575.0	(ID50)<br>0.4 J/cm²	U87MG	-	[14]
**Chlorin 5**	646 (EtOH)	1272.9	50	T98G	>10	[15]
**TPC–Ahx–ATWLPPR**	420 (EtOH)	-	171	HUVEC	-	[16]
**Acridine Orange**	487	265.4	-	U-373	-	[17]
**Porphyrazines (Pz I–IV)**	~600	1119.2<br>1023.1<br>1215.2<br>1003.2	1.18<br>0.56<br>0.54<br>0.39	GL261	-	[18,19]
**β-M-Ce6**	656	893.1	0.03	U251	-	[20]
**β-G-Ce6**	656	893.1	0.021	U251	-	[20]
**Thienopyrimidine derivative**	330 (MeOH)	431.2	5.0	F98	4	[21]
**ZnPc**<br>**TAZnPc**	645<br>685	577.9<br>637.9	Not specified	T98G<br>MO59<br>LN229<br>U87MG	-	[22]
**Sinoporphyrin sodium (DVDMS)**	631	1230.3	Not specified	U118MG<br>U87MG	-	[23]
**Chlorin derivative (ETPA)**	663	540.7	0.49	C6	204	[24]

**Rutherrin**	525	1007.1	(LD_{50})<br>2.39×10^{16}<br>hv/cm³	RG-2	-	25
**[Ru(η6 -p-cymene)(L1)Cl]**<br>**[Ru(η6 -p-cymene)(L2)Cl]**	447<br>461	518.2<br>568.2	22.8<br>21.7	LN229	-	26
**TMPC**	653	1077.5	(J/cm²)<br>18.39<br>26.47<br>44.79<br>83.02<br>161.67	U87	-	27
**Cercosporin**	473	534.5	(LD_{50})<br>4<br>4	U87MG<br>T98G	-	28
**THPTS**	760	1367.7	(IC_{30})<br>2<br>10<br>4	U87MG<br>A172<br>DBTRG-05	-	29
**Tetrakis(fluorophenyl) chlorin derivative**	652	1384.2	~0.001<br>~0.001<br>~0.001	U87MG<br>U251<br>T98G	-	30
**TLD1411**<br>**TLD1433**	416<br>416	950.9<br>936.1	0.37<br>0.46<br>0.051<br>2.81	U87MG<br>F98<br>U87MG<br>F98	280<br>397<br>3945<br>54	31
**HPPH-CD**<br>**$HPPH_2$-CD**	848<br>850	1555.7<br>2266.1	1.63<br>0.64	U87MG	-	32
**NSeMorph**	671	415.4	15.8<br>8.0	U87MG<br>U118MG	>31<br>>46.7	**This work**
**NSeAze**	671	355.3	0.38<br>0.46	U87MG<br>U118MG	>270<br>>245	**This work**

* measured in PBS (pH 7.4)
IC_{50} = half-maximal inhibitory concentrations
EC_{50} = half-maximal effective concentrations
ID_{50} = half-maximal irradiation dose to kill cells
LD_{50} = half-maximal lethal dose to kill population

NMR Spectra

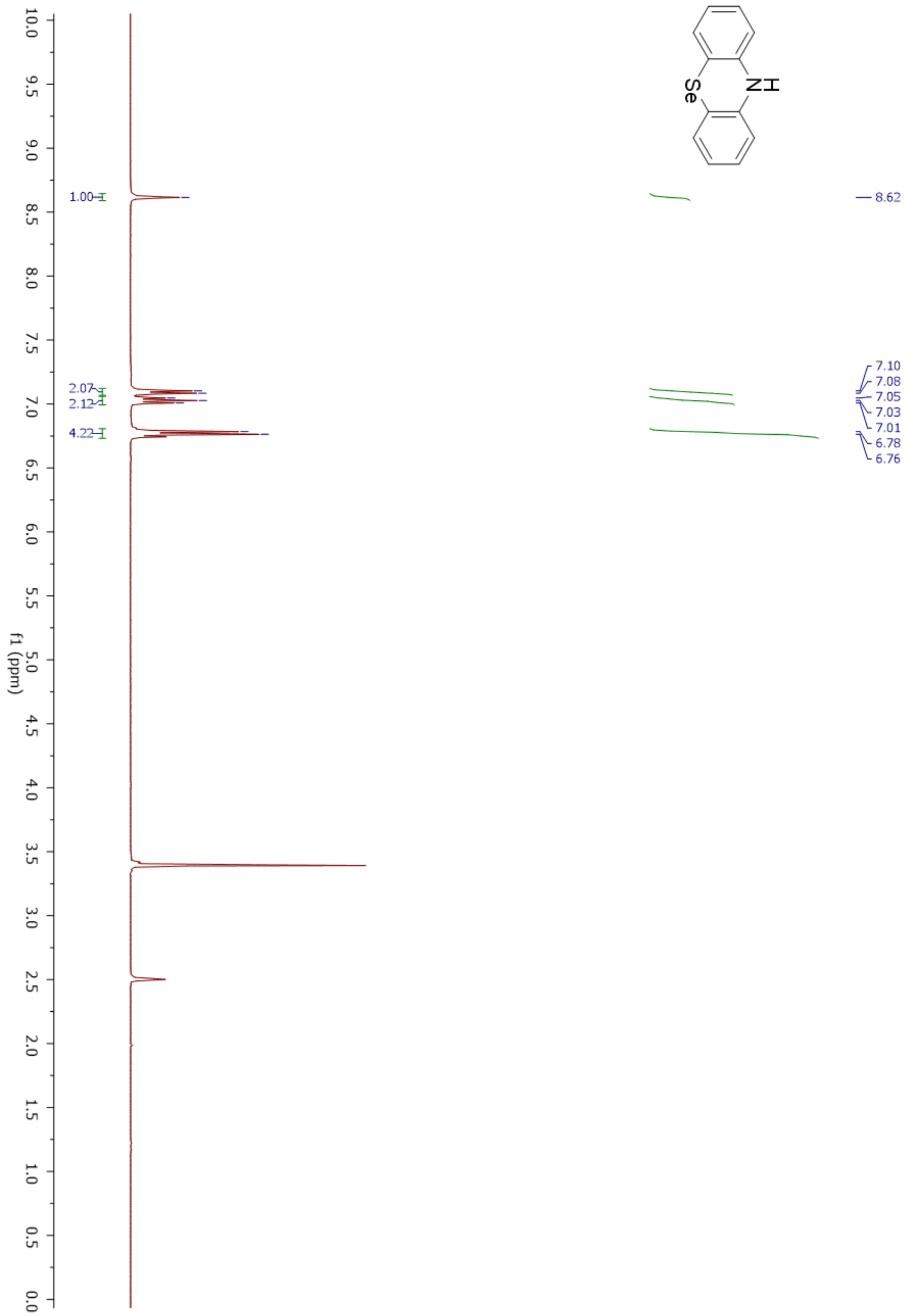

Figure S16. ^{1}H NMR spectrum of compound **2** in *d*-DMSO.

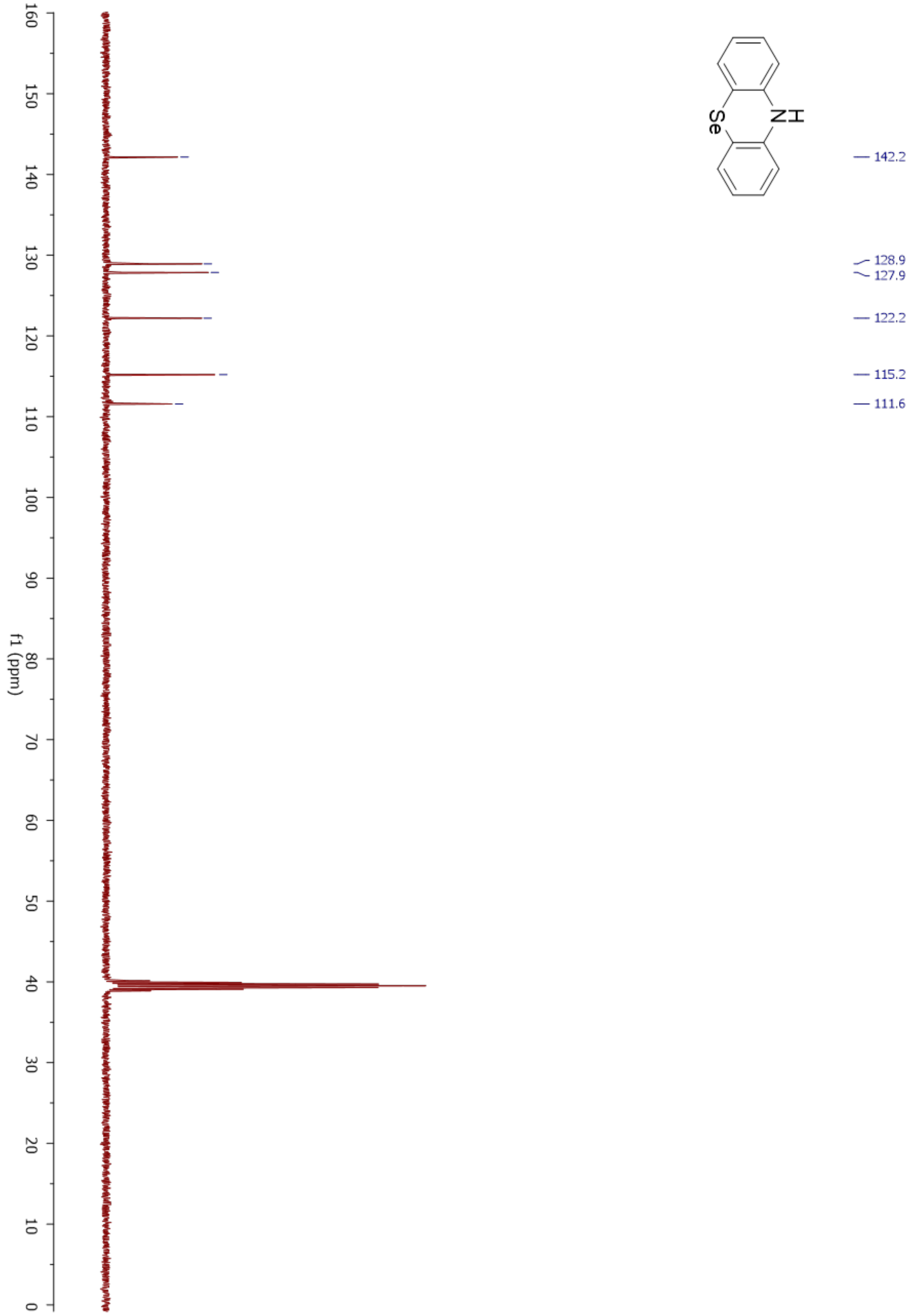

Figure S17. ^{13}C NMR spectrum of compound **2** in *d*-DMSO.

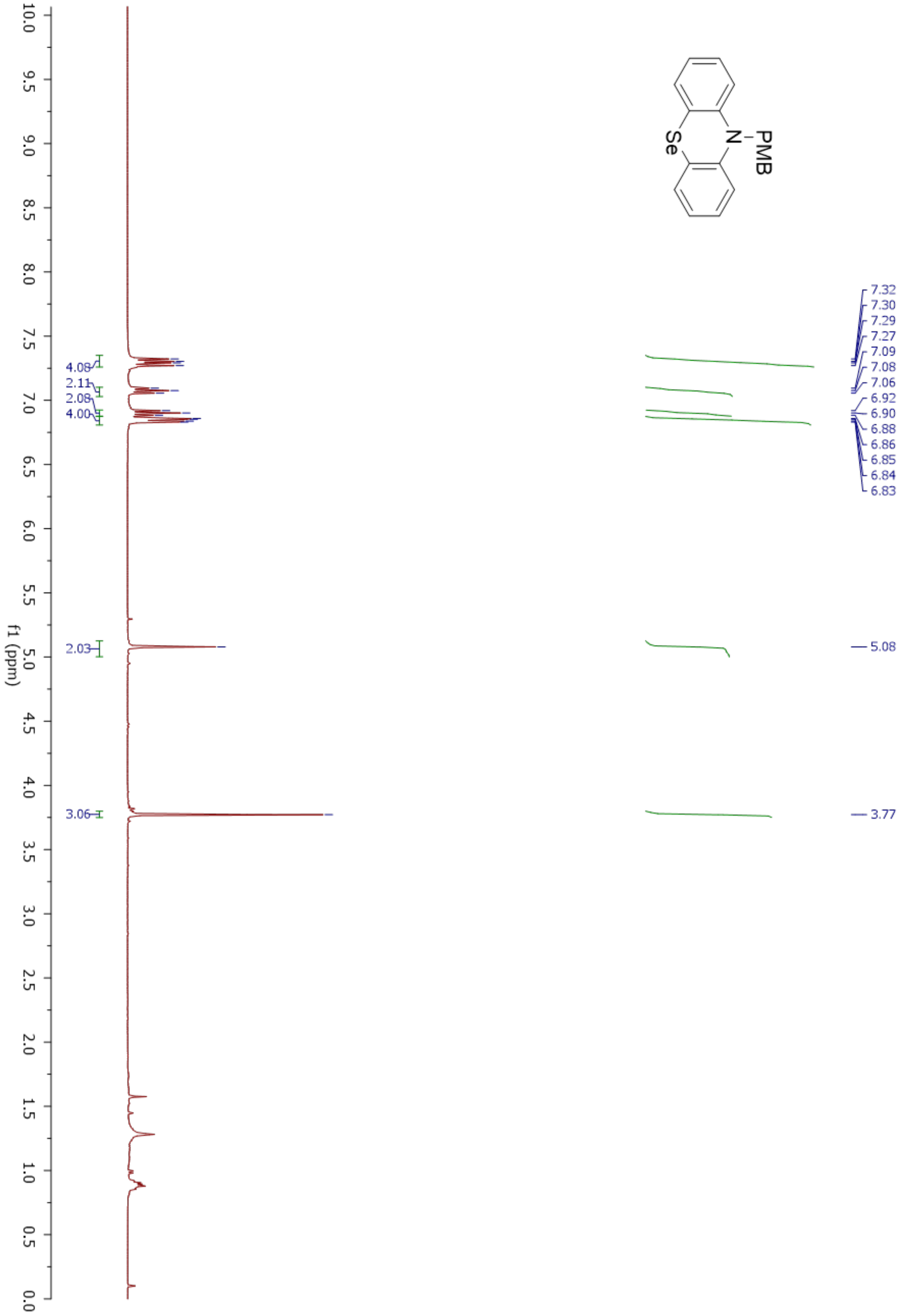

Figure S18. ^{1}H NMR spectrum of compound **3** in $CDCl_3$.

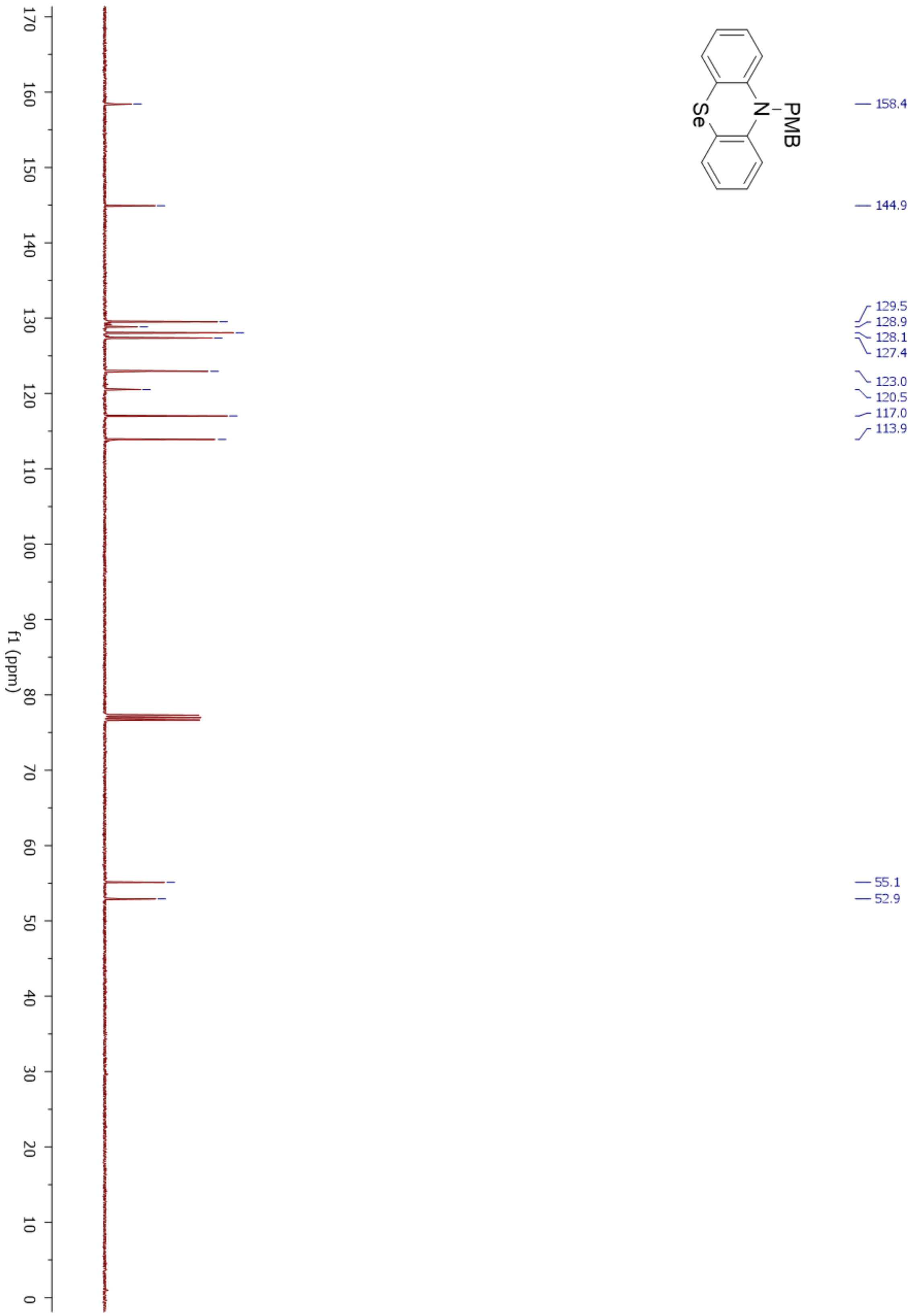

Figure S19. ^{13}C NMR spectrum of compound **3** in $CDCl_3$.

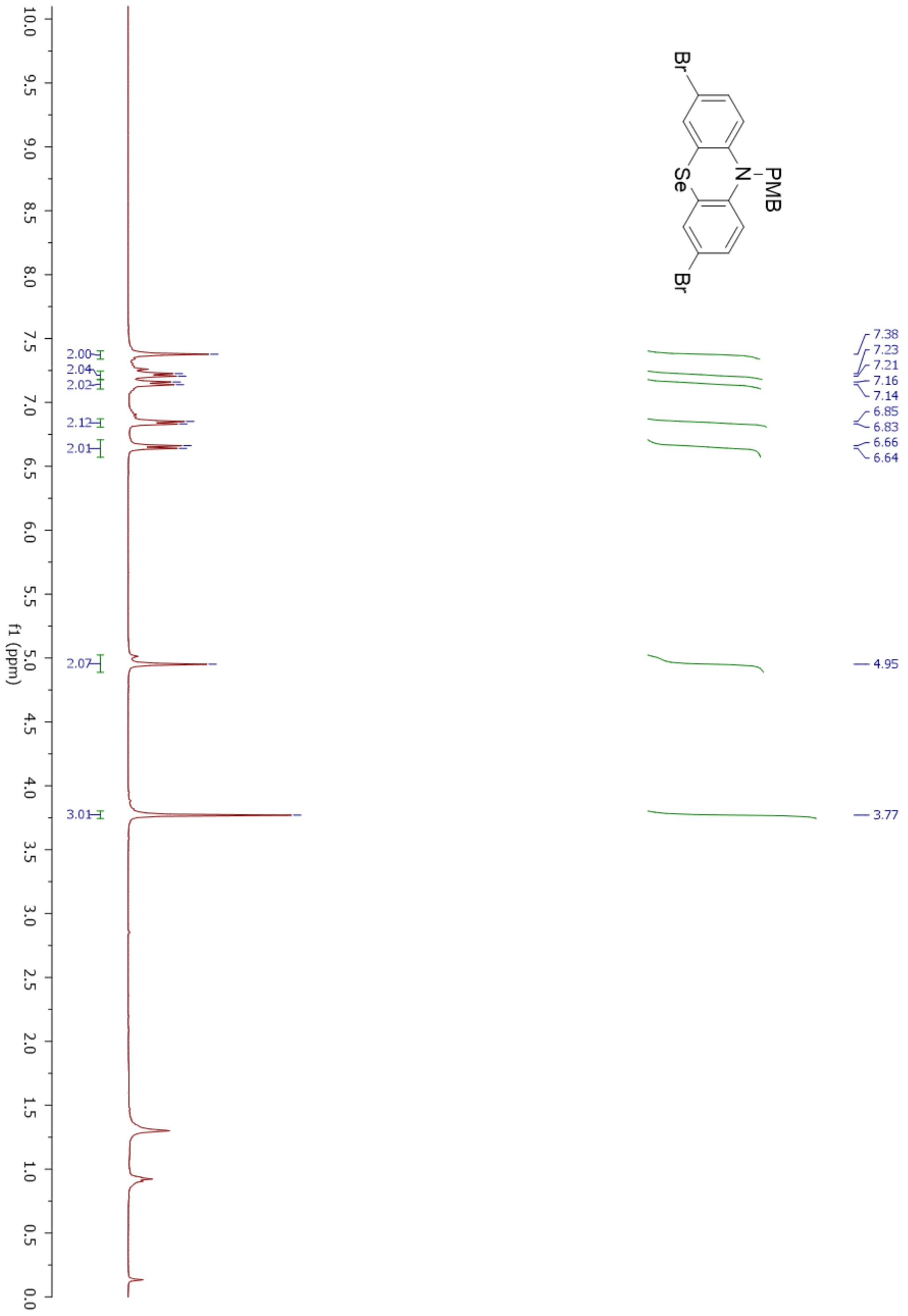

Figure S20. ^{1}H NMR spectrum of compound **4** in $CDCl_3$.

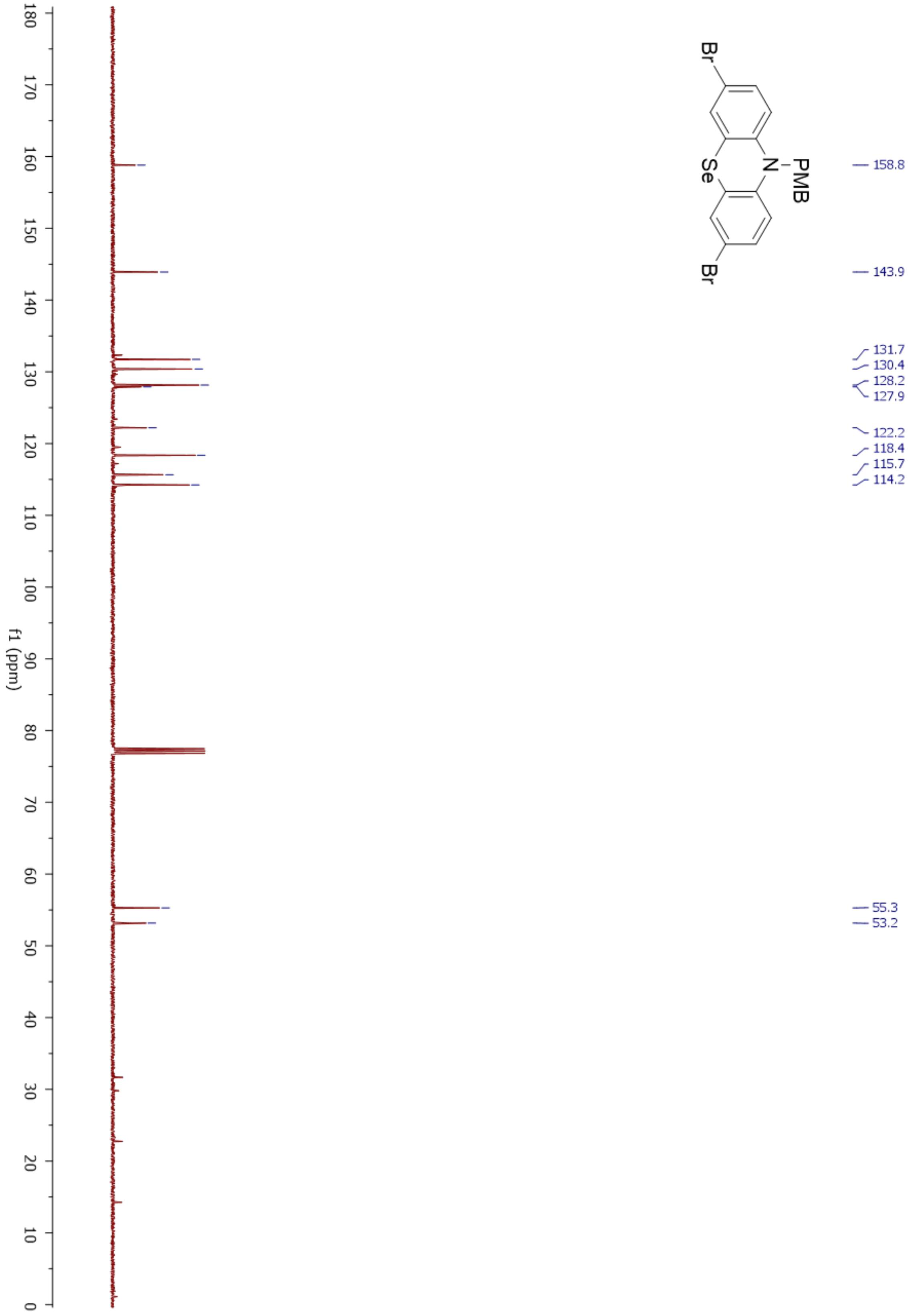

Figure S21. ^{13}C NMR spectrum of compound **4** in $CDCl_3$.

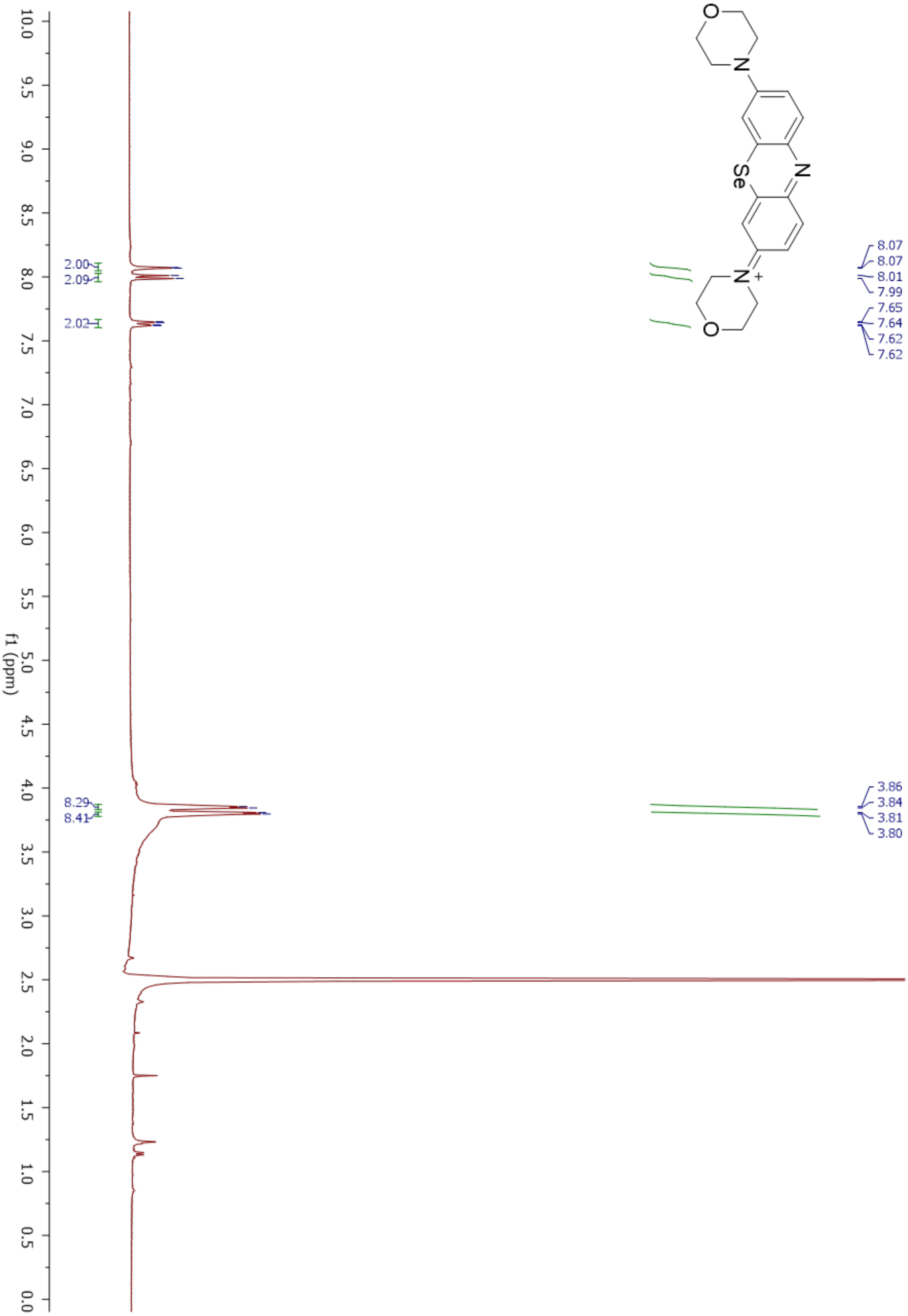

Figure S22. ^{1}H NMR spectrum of **NSeMorph** in *d*-DMSO.

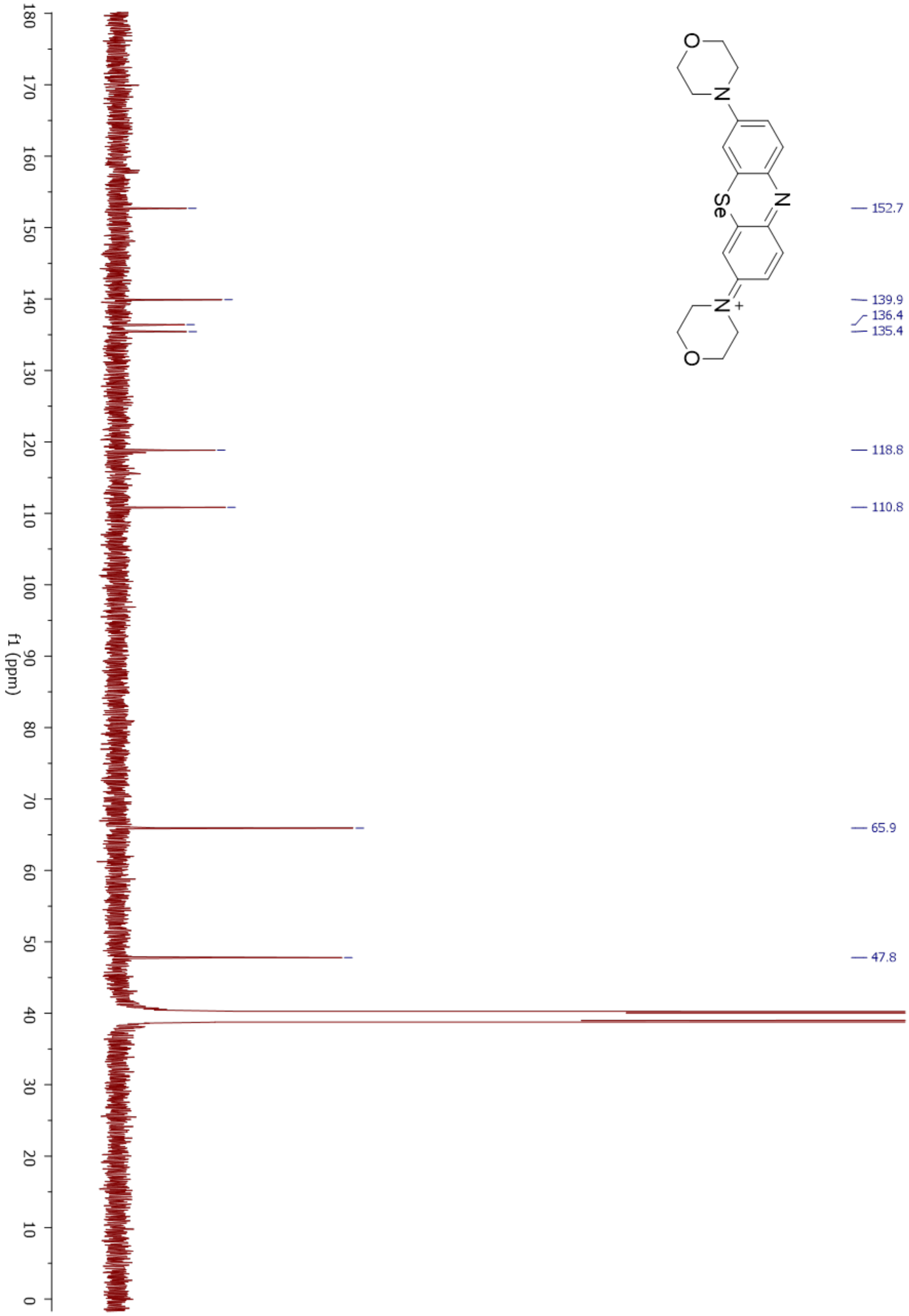

Figure S23. ^{13}C NMR spectrum of **NSeMorph** in *d*-DMSO.

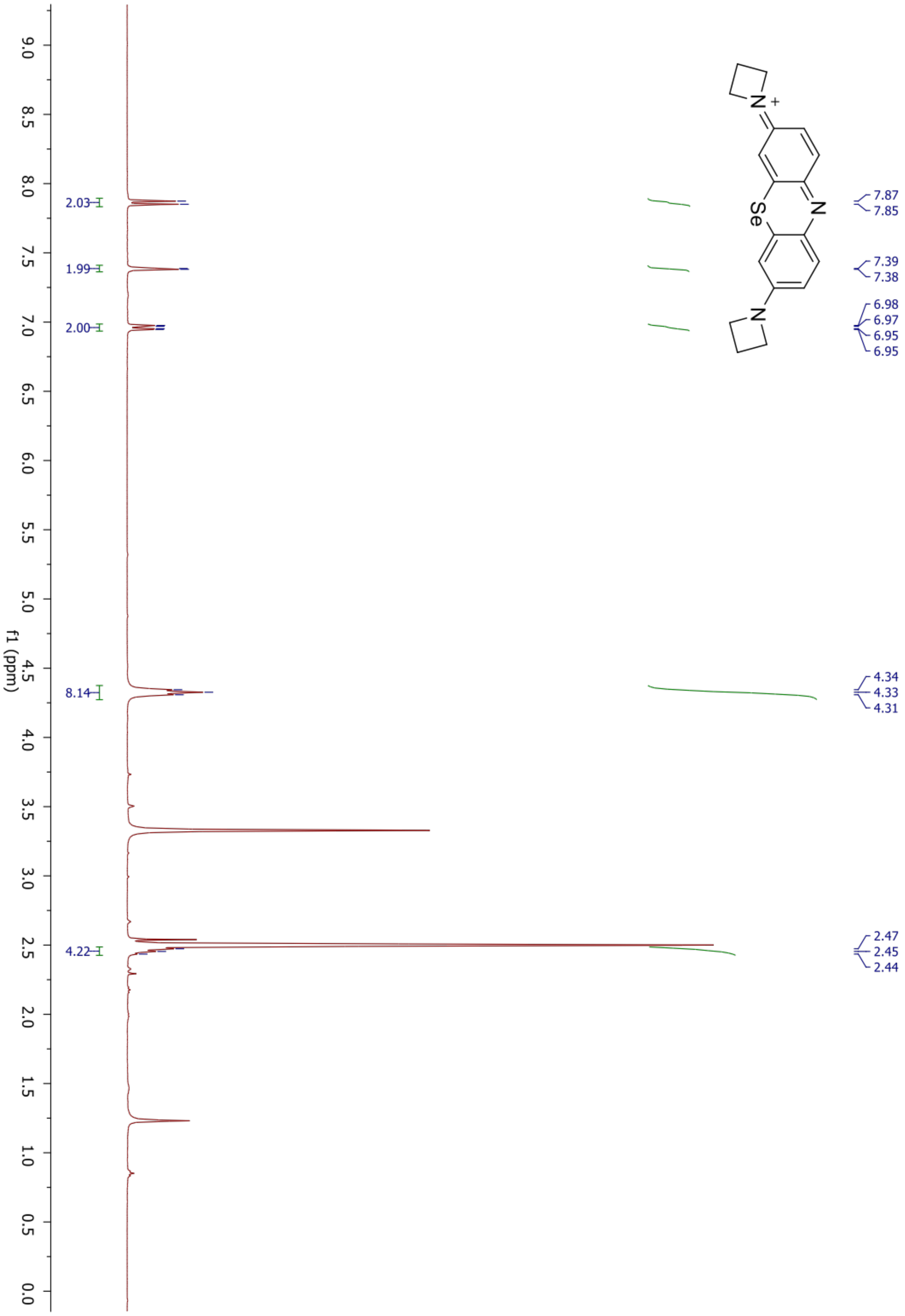

Figure S24. ^{1}H NMR spectrum of **NSeAze** in *d*-DMSO

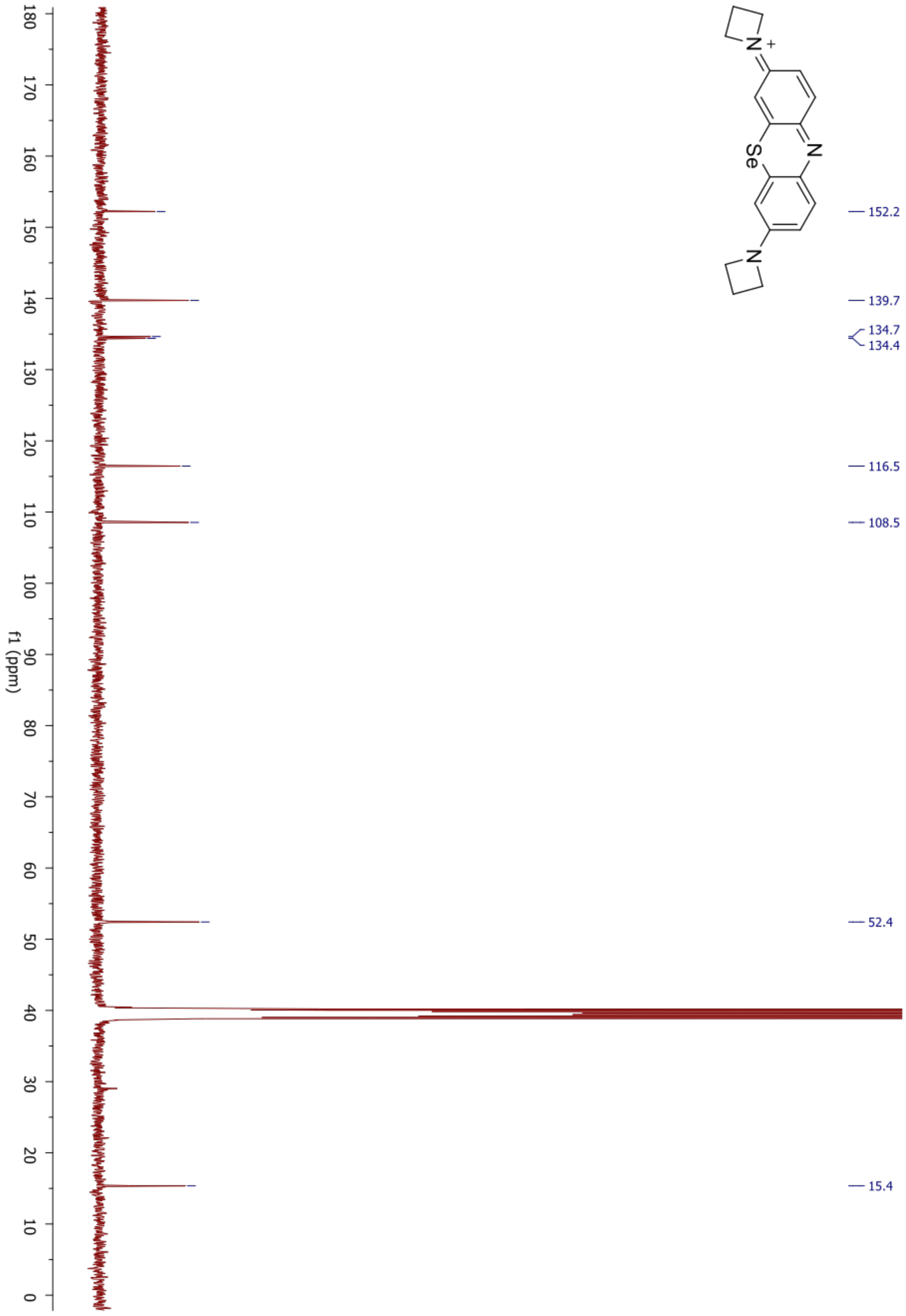

Figure S25. ^{13}C NMR spectrum of **NSeAze** in *d*-DMSO.

HRMS Results

Monoisotopic Mass, Odd and Even Electron Ions
1 formula(e) evaluated with 1 results within limits (all results (up to 1000) for each mass)
Elements Used:
C: 20-20 H: 17-18 N: 1-1 O: 1-1 Se: 1-1
Osman Karaman
31816_20210224_02-04 21 (0.829) Cm (11:24)

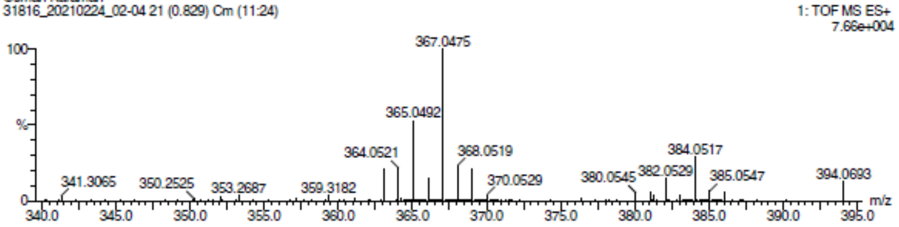

Minimum:				-5.5			
Maximum:		1000.0	1000.0	1000.0			
Mass	Calc. Mass	mDa	PPM	DBE	i-FIT	i-FIT (Norm)	Formula
367.0475	367.0475	0.0	0.0	14.0	479.1	0.0	C20 H17 N O Se

Figure S26. HRMS of compound **3.**

Monoisotopic Mass, Odd and Even Electron Ions
7 formula(e) evaluated with 1 results within limits (all results (up to 1000) for each mass)
Elements Used:
C: 20-20 H: 15-16 N: 1-1 O: 1-1 Se: 1-1 79Br: 0-2 81Br: 0-2

Osman Karaman
31816_20210224_03-02 12 (0.484) Cm (5:12)

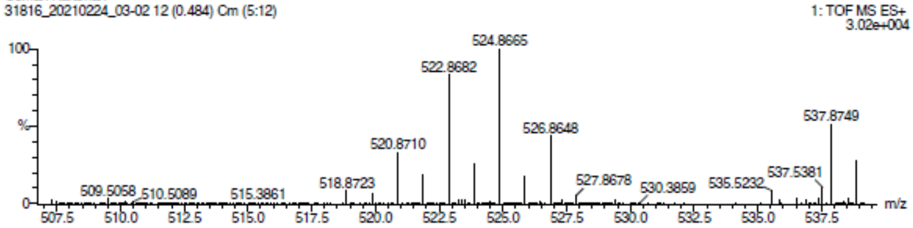

Minimum:				-5.5			
Maximum:		1000.0	1000.0	1000.0			
Mass	Calc. Mass	mDa	PPM	DBE	i-FIT	i-FIT (Norm)	Formula
524.8665	524.8665	0.0	0.0	14.0	327.6	0.0	C20 H15 N O Se 79Br 81Br

Figure S27. HRMS of compound **4.**

Monoisotopic Mass, Even Electron Ions
1 formula(e) evaluated with 1 results within limits (all results (up to 1000) for each mass)
Elements Used:
C: 20-20 H: 22-23 N: 3-3 O: 2-2 Se: 1-1
Osman Karaman
31816_20210224_04-03 10 (0.396) Cm (2:25)

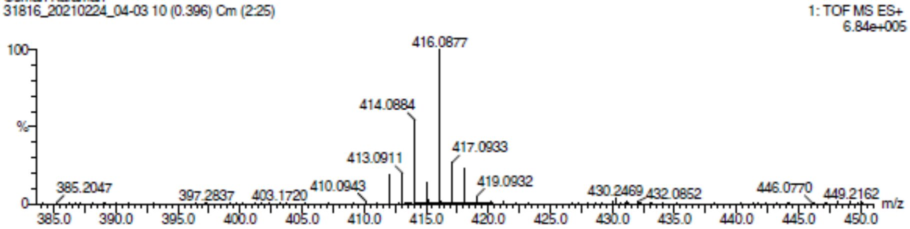

Minimum:				-5.5			
Maximum:		1000.0	1000.0	1000.0			
Mass	Calc. Mass	mDa	PPM	DBE	i-FIT	i-FIT (Norm)	Formula
416.0877	416.0877	0.0	0.0	12.5	747.2	0.0	C20 H22 N3 O2 Se

Figure S28. HRMS of **NSeMorph.**

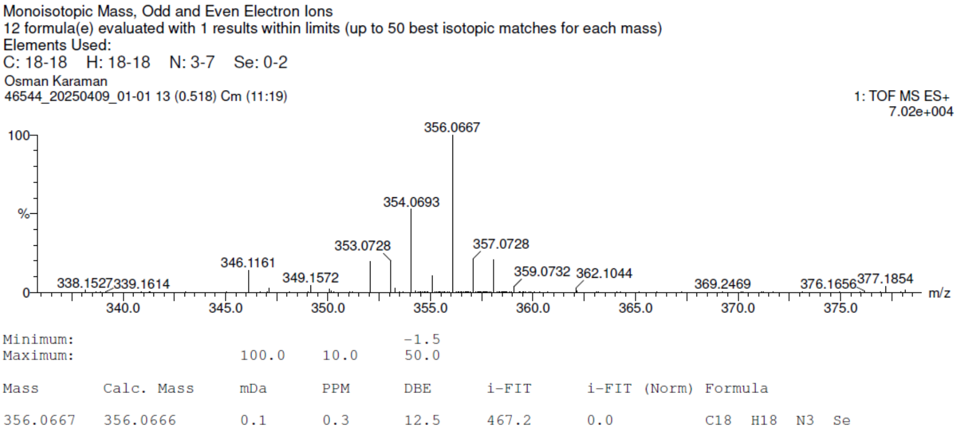

Figure S29. HRMS of **NSeAze.**

References

(1) Reyes-Soto, C. Y.; Ramírez-Carreto, R. J.; Ortíz-Alegría, L. B.; Silva-Palacios, A.; Zazueta, C.; Galván-Arzate, S.; Karasu, Ç.; Túnez, I.; Tinkov, A. A.; Aschner, M.; López-Goerne, T.; Anahí-Chavarría; Santamaría, A. S-Allyl-Cysteine Triggers Cytotoxic Events in Rat Glioblastoma RG2 and C6 Cells and Improves the Effect of Temozolomide through the Regulation of Oxidative Responses. *Discover Oncology* **2024**, *15* (1), 1–16. https://doi.org/10.1007/s12672-024-01145-3.

(2) Bailey, L. S.; Prajapati, D. V.; Basso, K. B. Optimization of the Sulfo-Phospho-Vanillin Assay for Total Lipid Normalization in Untargeted Quantitative Lipidomic LC-MS/MS Applications. *Anal Chem* **2022**, *94* (51), 17810–17818. https://doi.org/10.1021/acs.analchem.2c03488.

(3) Visnovitz, T.; Osteikoetxea, X.; Sódar, B. W.; Mihály, J.; Lőrincz, P.; Vukman, K. V.; Tóth, E. Á.; Koncz, A.; Székács, I.; Horváth, R.; Varga, Z.; Buzás, E. I. An Improved 96 Well Plate Format Lipid Quantification Assay for Standardisation of Experiments with Extracellular Vesicles. *J Extracell Vesicles* **2019**, *8* (1). https://doi.org/10.1080/20013078.2019.1565263.

(4) Chen, D. T. H.; Huang, S. Y.; Liu, T. C.; Chen, Y. K. Novel Compounds of Taiwanese Green Propolis Induce Apoptosis of Human Glioblastoma Cells by Daylight Photodynamic Action. *Future Sci OA* **2025**, *11* (1), 1–9. https://doi.org/10.1080/20565623.2025.2464491.

(5) Howley, R.; Olsen, J.; Chen, B. Effectiveness of Lapatinib for Enhancing 5-Aminolevulinic Acid-Mediated Protoporphyrin IX Fluorescence and Photodynamic Therapy in Human Cancer Cell Lines with Varied ABCG2 Activities. *Photochem Photobiol* **2024**, *100*, 1579–1589. https://doi.org/10.1111/php.13936.

(6) Jiang, Y.; Huang, S.; Ma, H.; Weng, J.; Du, X.; Lin, Z.; Kim, J.; You, W.; Zhang, H.; Wang, D.; Kim, J. S.; Sun, H. RNA-Activatable Near-Infrared Photosensitizer for Cancer Therapy. *J Am Chem Soc* **2024**, *146*, 25270–25281. https://doi.org/10.1021/jacs.4c09470.

(7) Wang, X.; Gao, Y.; Wang, T.; Wang, Z.; Hang, H.; Li, S.; Feng, F. Photoactivated Hydride Therapy under Hypoxia beyond ROS. *Chem Sci* **2024**, *15*, 20292–20302. https://doi.org/10.1039/d4sc06576j.

(8) Silva, M. J. S. A.; Zhang, Y.; Vinck, R.; Santos, F. M. F.; António, J. P. M.; Gourdon-Grünewaldt, L.; Zaouter, C.; Castonguay, A.; Patten, S. A.; Cariou, K.; Boscá, F.; Nájera, F.; Arteaga, J. F.; Gasser, G.; Pischel, U.; Gois, P. M. P. BASHY Dyes Are Highly Efficient Lipid Droplet-Targeting Photosensitizers That Induce Ferroptosis through Lipid Peroxidation. *Bioconjug Chem* **2023**, *34* (12), 2337–2344. https://doi.org/10.1021/acs.bioconjchem.3c00449.

(9) Teja, H. B.; Bhojya Naik, H. S.; Amith Nayak, P. H.; Prabhakara, M. C. Study of Photophysico-Chemical and Antimicrobial Photodynamic Properties of Visible Light Active Azometal(Ii) Complexes. *Asian Journal of Chemistry* **2021**, *33* (8), 1709–1717. https://doi.org/10.14233/ajchem.2021.23178.

(10) Baglo, Y.; Sorrin, A. J.; Liang, B. J.; Huang, H. C. Harnessing the Potential Synergistic Interplay Between Photosensitizer Dark Toxicity and Chemotherapy. *Photochem Photobiol* **2020**, *96* (3), 636–645. https://doi.org/10.1111/php.13196.

(11) Kielbik, A.; Wawryka, P.; Przystupski, D.; Rossowska, J.; Szewczyk, A.; Saczko, J.; Kulbacka, J.; Chwiłkowska, A. Effects of Photosensitization of Curcumin in Human Glioblastoma Multiforme Cells. *In Vivo (Brooklyn)* **2019**, *33* (6), 1857–1864. https://doi.org/10.21873/invivo.11679.

(12) Liu, Q.; Pang, M.; Tan, S.; Wang, J.; Chen, Q.; Wang, K.; Wu, W.; Hong, Z. Potent Peptide-Conjugated Silicon Phthalocyanines for Tumor Photodynamic Therapy. *J Cancer* **2018**, *9* (2), 310–320. https://doi.org/10.7150/jca.22362.

(13) Zhu, J.; Dominijanni, A.; Rodríguez-Corrales, J.; Prussin, R.; Zhao, Z.; Li, T.; Robertson, J. L.; Brewer, K. J. Visible Light-Induced Cytotoxicity of Ru,Os–Polyazine Complexes towards Rat Malignant Glioma. *Inorganica Chim Acta* **2017**, *454*, 155–161. https://doi.org/10.1016/j.ica.2016.05.044.

(14) Silva, E. P. O.; Franchi, L. P.; Tedesco, A. C. Chloro-Aluminium Phthalocyanine Loaded in Ultradeformable Liposomes for Photobiology Studies on Human Glioblastoma. *RSC Adv* **2016**, *6* (83), 79631–79640. https://doi.org/10.1039/c6ra16015h.

(15) Luguya, R.; Jensen, T. J.; Smith, K. M.; Vicente, M. G. H. Synthesis and Cellular Studies of a Carboranylchlorin for the PDT and BNCT of Tumors. *Bioorg Med Chem* **2006**, *14* (17), 5890–5897. https://doi.org/10.1016/j.bmc.2006.05.026.

(16) Tirand, L.; Frochot, C.; Vanderesse, R.; Thomas, N.; Trinquet, E.; Pinel, S.; Viriot, M. L.; Guillemin, F.; Barberi-Heyob, M. A Peptide Competing with VEGF165 Binding on Neuropilin-1 Mediates Targeting of a Chlorin-Type Photosensitizer and Potentiates Its Photodynamic Activity in Human Endothelial Cells. *Journal of Controlled Release* **2006**, *111* (1–2), 153–164. https://doi.org/10.1016/j.jconrel.2005.11.017.

(17) Osman, H.; Elsahy, D.; Saadatzadeh, M. R.; Pollok, K. E.; Yocom, S.; Hattab, E. M.; Georges, J.; Cohen-Gadol, A. A. Acridine Orange as a Novel Photosensitizer for Photodynamic Therapy in Glioblastoma. *World Neurosurg* **2018**, *114*, 1310–1315. https://doi.org/10.1016/j.wneu.2018.03.207.

(18) Mishchenko, T. A.; Turubanova, V. D.; Mitroshina, E. V.; Alzeibak, R.; Peskova, N. N.; Lermontova, S. A.; Klapshina, L. G.; Balalaeva, I. V.; Vedunova, M. V.; Krysko, D. V. Effect of Novel Porphyrazine Photosensitizers on Normal and Tumor Brain Cells. *J Biophotonics* **2020**, *13* (1), 1–9. https://doi.org/10.1002/jbio.201960077.

(19) Redkin, T. S.; Sleptsova, E. E.; Turubanova, V. D.; Saviuk, M. O.; Lermontova, S. A.; Klapshina, L. G.; Peskova, N. N.; Balalaeva, I. V.; Krysko, O.; Mishchenko, T. A.; Vedunova, M. V.; Krysko, D. V. Dendritic Cells Pulsed with Tumor Lysates Induced by Tetracyanotetra(Aryl)Porphyrazines-Based Photodynamic Therapy Effectively Trigger Anti-Tumor Immunity in an Orthotopic Mouse Glioma Model. *Pharmaceutics* **2023**, *15* (10), 2430–2447. https://doi.org/10.3390/pharmaceutics15102430.

(20) Nishie, H.; Kataoka, H.; Yano, S.; Yamaguchi, H.; Nomoto, A.; Tanaka, M.; Kato, A.; Shimura, T.; Mizoshita, T.; Kubota, E.; Tanida, S.; Joh, T. Excellent Antitumor Effects for Gastrointestinal Cancers Using Photodynamic Therapy with a Novel Glucose Conjugated Chlorin E6. *Biochem Biophys Res Commun* **2018**, *496* (4), 1204–1209. https://doi.org/10.1016/j.bbrc.2018.01.171.

(21) Gederaas, O. A.; Sørensen, A. S.; Lindgren, M.; Melø, T. B.; Altin, D.; Flatby, E. M.; Høgset, A.; Hoff, B. H. Synthesis and in Vitro Evaluation of a Novel Thienopyrimidine with Phototoxicity towards Rat Glioma F98 Cells. *J Photochem Photobiol* **2022**, *10*, 1–8. https://doi.org/10.1016/j.jpap.2022.100114.

(22) Velazquez, F. N.; Miretti, M.; Baumgartner, M. T.; Caputto, B. L.; Tempesti, T. C.; Prucca, C. G. Effectiveness of ZnPc and of an Amine Derivative to Inactivate Glioblastoma Cells by Photodynamic Therapy: An in Vitro Comparative Study. *Sci Rep* **2019**, *9* (1), 1–15. https://doi.org/10.1038/s41598-019-39390-0.

(23) An, Y. W.; Liu, H. Q.; Zhou, Z. Q.; Wang, J. C.; Jiang, G. Y.; Li, Z. W.; Wang, F.; Jin, H. T. Sinoporphyrin Sodium Is a Promising Sensitizer for Photodynamic and Sonodynamic Therapy in Glioma. *Oncol Rep* **2020**, *44* (4), 1596–1604. https://doi.org/10.3892/or.2020.7695.

(24) Klimenko, A.; Rodina, E. E.; Silachev, D.; Begun, M.; Babenko, V. A.; Benditkis, A. S.; Kozlov, A. S.; Krasnovsky, A. A.; Khotimchenko, Y. S.; Katanaev, V. L. Chlorin Endogenous to the North Pacific Brittle Star Ophiura Sarsii for Photodynamic Therapy Applications in Breast Cancer and Glioblastoma Models. *Biomedicines* **2022**, *10* (134), 1–13. https://doi.org/10.3390/biomedicines10010134.

(25) Munegowda, M. A.; Fisher, C.; Molehuis, D.; Foltz, W.; Roufaiel, M.; Bassan, J.; Nitz, M.; Mandel, A.; Lilge, L. Efficacy of Ruthenium Coordination Complex–Based Rutherrin in a Preclinical Rat Glioblastoma Model. *Neurooncol Adv* **2019**, *1* (1), 1–12. https://doi.org/10.1093/noajnl/vdz006.

(26) Kumar, P.; Mondal, I.; Kulshreshtha, R.; Patra, A. K. Development of Novel Ruthenium(Ii)-Arene Complexes Displaying Potent Anticancer Effects in Glioblastoma Cells. *Dalton Transactions* **2020**, *49* (38), 13294–13310. https://doi.org/10.1039/d0dt02167a.

(27) Wang, L. X.; Li, J. W.; Huang, J. Y.; Li, J. H.; Zhang, L. J.; O'Shea, D.; Chen, Z. L. Antitumor Activity of Photodynamic Therapy with a Chlorin Derivative in Vitro

and in Vivo. *Tumor Biology* **2015**, *36* (9), 6839–6847. https://doi.org/10.1007/s13277-015-3395-1.

(28) Mastrangelopoulou, M.; Grigalavicius, M.; Berg, K.; Ménard, M.; Theodossiou, T. A. Cytotoxic and Photocytotoxic Effects of Cercosporin on Human Tumor Cell Lines. *Photochem Photobiol* **2019**, *95* (1), 387–396. https://doi.org/10.1111/php.12997.

(29) Hambsch, P.; Istomin, Y. P.; Tzerkovsky, D. A.; Patties, I.; Neuhaus, J.; Kortmann, R.-D.; Schastak, S.; Glasow, A. Efficient Cell Death Induction in Human Glioblastoma Cells by Photodynamic Treatment with Tetrahydroporphyrin-Tetratosylat (THPTS) and Ionizing Irradiation. *Oncotarget* **2017**, *8* (42), 72411–72423.

(30) Hirohara, S.; Oka, C.; Totani, M.; Obata, M.; Yuasa, J.; Ito, H.; Tamura, M.; Matsui, H.; Kakiuchi, K.; Kawai, T.; Kawaichi, M.; Tanihara, M. Synthesis, Photophysical Properties, and Biological Evaluation of Trans-Bisthioglycosylated Tetrakis(Fluorophenyl)Chlorin for Photodynamic Therapy. *J Med Chem* **2015**, *58* (21), 8658–8670. https://doi.org/10.1021/acs.jmedchem.5b01262.

(31) Fong, J.; Kasimova, K.; Arenas, Y.; Kaspler, P.; Lazic, S.; Mandel, A.; Lilge, L. A Novel Class of Ruthenium-Based Photosensitizers Effectively Kills in Vitro Cancer Cells and in Vivo Tumors. *Photochemical and Photobiological Sciences* **2015**, *14* (11), 2014–2023. https://doi.org/10.1039/c4pp00438h.

(32) James, N. S.; Ohulchanskyy, T. Y.; Chen, Y.; Joshi, P.; Zheng, X.; Goswami, L. N.; Pandey, R. K. Comparative Tumor Imaging and PDT Efficacy of HPPH Conjugated in the Mono-and Di-Forms to Various Polymethine Cyanine Dyes: Part-2. *Theranostics* **2013**, *3* (9), 703–718. https://doi.org/10.7150/thno.5923.
